# Gas7 Is a Novel Dendritic Spine Initiation Factor

**DOI:** 10.1523/ENEURO.0344-22.2023

**Published:** 2023-04-13

**Authors:** Pushpa Khanal, Zoran Boskovic, Lauri Lahti, Aruna Ghimire, Rimante Minkeviciene, Patricio Opazo, Pirta Hotulainen

**Affiliations:** 1 Minerva Foundation Institute for Medical Research, Helsinki 00290, Finland; 2 HiLIFE-Neuroscience Center, University of Helsinki, Helsinki 00290, Finland; 3 Clem Jones Centre for Ageing Dementia Research, The Queensland Brain Institute, The University of Queensland, Brisbane, Queensland, 4072, St Lucia, Australia; 4 Department of Computer Science, Aalto University School of Science, 00076 Espoo, Finland; 5 Centre for Discovery Brain Sciences, United Kingdom Dementia Research Institute at the University of Edinburgh, Edinburgh EH8 9XD, United Kingdom

**Keywords:** actin cytoskeleton, BAR domains, dendritic spines, spine initiation

## Abstract

Brain stores new information by modifying connections between neurons. When new information is learnt, a group of neurons gets activated and they are connected to each other via synapses. Dendritic spines are protrusions along neuronal dendrites where excitatory synapses are located. Dendritic spines are the first structures to protrude out from the dendrite to reach out to other neurons and establish a new connection. Thus, it is expected that neuronal activity enhances spine initiation. However, the molecular mechanisms linking neuronal activity to spine initiation are poorly known. Membrane binding BAR domain proteins are involved in spine initiation, but it is not known whether neuronal activity affects BAR domain proteins. Here, we used bicuculline treatment to activate excitatory neurons in organotypic hippocampal slices. With this experimental setup, we identified F-BAR domain containing growth arrest-specific protein (Gas7) as a novel spine initiation factor responding to neuron activity. Upon bicuculline addition, Gas7 clustered to create spine initiation hotspots, thus increasing the probability to form new spines in activated neurons. Gas7 clustering and localization was dependent on PI3-kinase (PI3K) activity and intact F-BAR domain. Gas7 overexpression enhanced N-WASP localization to clusters as well as it increased the clustering of actin. Arp2/3 complex was required for normal Gas7-induced actin clustering. Gas7 overexpression increased and knock-down decreased spine density in hippocampal pyramidal neurons. Taken together, we suggest that Gas7 creates platforms under the dendritic plasma membrane which facilitate spine initiation. These platforms grow on neuronal activation, increasing the probability of making new spines and new connections between active neurons. As such, we identified a novel molecular mechanism to link neuronal activity to the formation of new connections between neurons.

## Significance Statement

When we learn something new, neurons change their connections to other neurons. We know that active neurons connect to each other, but we have poor understanding on the cellular or molecular mechanisms which link neuronal activity to the formation of new connections. Here we show how neuronal activation can facilitate formation of new connections to wire the firing neurons together.

## Introduction

Recent findings indicate that memory storage processes operate conjointly at the level of neurons, dendrites, and dendritic spines. The group of neurons that store a specific information together is called an engram (Ortega-de San Luis and Ryan, 2022). Neuronal network contains many engrams and different engrams can use the same cells and same synapses, but the combination in which they are used is crucial (Cai et al., 2016). Allocation of neurons to an engram depends on their activity, and in a simplified view, active neurons connect to each other and then later get activated together (Jeong et al., 2021). Based on these ideas, it is expected that neuronal activation would enhance formation of connections between active neurons. However, we have a poor understanding of how neuronal activity could enhance the formation of connections at the molecular level.

Dendritic spines are small, dynamic, actin-rich protrusions along the neuronal dendrites where most of the excitatory synapses are located. Size, morphology, and density of dendritic spines directly affect synaptic function and plasticity (Arellano et al., 2007; Holtmaat and Svoboda, 2009). The appearance of new spines has been associated with novel sensory experience and learning new tasks (Holtmaat et al., 2006; Hofer et al., 2009; Xu et al., 2009; Yang et al., 2009; Roberts et al., 2010). Initiation of new spines is a molecular mechanism to regulate the rate of spine formation and there is strong evidence that an increased neural activity enhances new spine growth (Papa and Segal, 1996; Engert and Bonhoeffer, 1999; Maletic-Savatic et al., 1999; Kwon and Sabatini, 2011; Chatzi et al., 2019). Multiple studies also demonstrate that activity-induced spine outgrowth is dependent on NMDA-receptor signaling (Engert and Bonhoeffer, 1999; Maletic-Savatic et al., 1999; Kwon and Sabatini, 2011). Hamilton et al. (2012) further showed that proteasomal degradation is necessary for activity-induced spine outgrowth (Hamilton et al., 2012).

In our previous study, we identified a protein called missing-in-metastasis (MIM/Mtss1) as a novel spine initiation factor (Saarikangas et al., 2015). Spine initiation factors are molecules which change the shape of plasma membrane to push a new protrusion out. Molecular mechanism can be, for example, directly curving the membrane (membrane curving proteins) or inducing membrane curvature through other means, such as directing actin polymerization toward plasma membrane. MIM/Mtss1 is an I-BAR-domain containing protein, known to curve the plasma membrane in such a way that it protrudes out forming a small protrusion on the cell edge. We showed that MIM/Mtss1 is important for achieving and maintaining normal spine density in the early development (Saarikangas et al., 2015). In addition to MIM/Mtss1, iF-BAR domain containing SrGAP3, is a spine initiation factor (Carlson et al., 2011). Furthermore, the expression of close MIM/Mtss1 homolog ABBA/Mtss1l was increased after physical exercise. Exercise also increased neuronal activity and formation of new spines in activated cells (Chatzi et al., 2019). This data supports the idea that ABBA/Mtss1l is also functioning as a spine initiation factor. Thus, we hypothesized that also other BAR domain proteins could be involved in spine initiation (Khanal and Hotulainen, 2021). However, it has not been tested experimentally which BAR domain proteins can facilitate spine initiation or whether neuronal activity affects BAR domain proteins.

Hamilton et al. (2012) used bicuculline treatment to activate neurons in organotypic slices and we followed the same experimental setup to test whether we can identify a BAR domain containing protein that would respond to synaptic activation. With this testing, we found a protein called growth arrest-specific protein (Gas7). Gas7 is an F-BAR domain containing protein, which binds PI(3,4,5)P3 (Hanawa-Suetsugu et al., 2019). Gas7 makes sheet like scaffolds under the plasma membrane (Hanawa-Suetsugu et al., 2019) and it directly binds N-WASP (You and Lin-Chao, 2010; Tseng et al., 2015). N-WASP regulates actin polymerization through Arp2/3 complex (Rohatgi et al., 1999). Here, we demonstrate that Gas7 is a novel spine initiation factor, which responds to neuronal activation. With these results we reveal a novel mechanism linking neuronal activity to spine initiation.

## Materials and Methods

### Plasmid construction

The plasmids pmCherry-C1 and pEGFP-C1 were purchased from the Clontech Laboratories Inc. shRNA constructs against rat Gas7 (NM_053484.2) were purchased from GeneCopoeia. For shRNA #1, clone name: RSH088545-23-mH1 and target sequence: GCTGCAGAAGCAACTGAAAGG, for shRNA #2, clone name: RSH088545-24-mH1 and target sequence: GGTAGAGATGATCCGACAACA, and for the control shRNA, clone name: CSHCTR001-2-mH1, and the target sequence: TGGCTGCATGCTATGTTGA.

The GFP and mCherry-tagged wild-type (WT) growth arrest-specific 7 (Gas7) were generated using the Genome Biology Unit core facility cloning service (Research Programs Unit, HiLIFE Helsinki Institute of Life Science, Faculty of Medicine, University of Helsinki, Biocenter Finland). Briefly, entry clone (clone-ID: 100005432) from the human ORFeome collaboration library was transferred into the pcDNA6.2/N-emGFP-DEST and N-mCherry, respectively, using the standard LR reaction protocol. The FBAR domain of Gas7 was PCR amplified from Gas7-pcDNA6.2/N-emGFP-DEST and cloned into the XhoI and BamHI of pEGFP-C1 and pmCherry-C1 using the following primers: forward: GGACTCAGATCTCGAAACGGCACCGTGGCTGGG and reverse: TAGATCCGGTGGATCCTACTGATCCACGGGCTCG.

The RFP-LifeAct and Ruby-LifeAct constructs were kind gifts from Roland Wedlich-Söldner (University of Münster, Münster, Germany; Riedl et al., 2008). The murine N-WASP mCherry construct was kindly provided by Maria Vartiainen (Institute of Biotechnology, University of Helsinki). The Scar W and WA constructs (Machesky and Insall, 1998) were a generous gift from Laura Machesky (Beatson Institute for Cancer Research, Glasgow, United Kingdom). Akt PH domain-mRFP construct was given by Vesa Olkkonen (Minerva Foundation Institute for Medical Research).

The mutant constructs were generated by PCR-mediated site-directed mutagenesis (Zheng et al., 2004; Hlushchenko et al., 2018). The PCR products were purified and treated with DpnI (New England Biolabs) for 2 h. Then, the 5 ml of DpnI-treated PCR product was used to transform the DH5-α cells. The transformants were inoculated and grown on LB plates with appropriate antibiotics. 5–10 colonies were selected for miniprep. The mutant constructs were then verified by DNA sequencing. The primers used for generating each mutant construct are listed in Table 1. The mutagenized nucleotides are shown in bold.

**Table 1:** Primers for mutation constructs

Template used	Mutation introduced	Primers
WT Gas7	Del (29 aa deletion from 167 to 195)	Forward: CAGCTGAAGGGCCAGAACTCCTTGGCTTC Reverse: GGAGTTCTGGCCCTTCAGCTGTTTCTGGAG
WT Gas7	mut1 (R178A)	Forward: GAATTCATCCGGGAA**GC**GATAAAGATTGAAG Reverse: CTTTATC**GC**TTCCCGGATGAATTCTGACATTTC
mut1	mut2 (R178A, R180A)	Forward: GAATTCATC**GC**GGAA**GC**GATAAAGATTGAAG Reverse: CTTTATC**GC**TTCC**GC**GATGAATTCTGACATTTC
mut2	mut3 (K171A, R178A, R180A)	Forward: GGCAAACAAATGCAG**GC**GGAAATGTCAG Reverse: GATGAATTCTGACATTTCC**GC**CTGCATTTG
WT Gas7	W19A	Forward: CACCTGGC**GC**GCAGAGCTACCTGTCGCCTCA Reverse: GTAGCTCTGC**GC**GCCAGGTGGAAGGATG
W19A	WAWA (W19A and W41A)	Forward: GAGACCACC**GC**GGAACGTCCCAGCAGTTCTCC Reverse: CTGGGGACGTTCC**GC**GGTGGTCTCATTGGTG

The mutagenized nucleotides are shown in bold.

**Table 2:** Summary of statistics

Figure		Data structure	Type of test	*p*-values
Fig. 1*B*		Nonparametric	Mann–Whitney *U* test	<0.001
Fig. 1*D*		Nonparametric	Wilcoxon paired test	<0.01
Fig. 1*F*		Nonparametric	Mann–Whitney *U* test	<0.05
Fig. 2*C*		Normally distributed	Unpaired *t* test	<0.01
Fig. 2*E*		Normally distributed	Unpaired *t* test	<0.001
Fig. 2*F*		Normally distributed	Unpaired *t* test	<0.001
Fig. 2*H*		Normally distributed	Unpaired *t* test	<0.001
Fig. 2*I*		Normally distributed	Unpaired *t* test	<0.001
Fig. 3*C*		Normally distributed	Unpaired *t* test	<0.001
Fig. 4*B*		Normally distributed	One-way ANOVA; Games–Howell *post hoc* test	<0.001
Fig. 4*D*		Normally distributed	One-way ANOVA; Bonferroni’s *post hoc* test	<0.001
Fig. 4E		Normally distributed	One-way ANOVA; Bonferroni’s *post hoc* test	<0.01 <0.001
Extended Data Fig. 4-1*B*		Normally distributed	One-way ANOVA; Bonferroni’s *post hoc* test	<0.01 <0.001
Extended Data Fig. 4-1*C*		Normally distributed/nonparametric	One-way ANOVA/Kruskal–Wallis test	<0.05 <0.01
Extended Data Fig. 4-1*D*		Normally distributed	One-way ANOVA; Games–Howell *post hoc* test	<0.01 <0.001
Extended Data Fig. 4-1*E*		Normally distributed/nonparametric	One-way ANOVA/Kruskal–Wallis test	<0.05 <0.01 <0.001
Fig. 5*C*		Nonparametric	Mann–Whitney *U* test	<0.01
Fig. 5*F*		Nonparametric	Wilcoxon paired test	<0.05
Fig. 6*B*		Nonparametric	Kruskal–Wallis test	<0.05 <0.01
Fig. 6*E*		Nonparametric	Mann–Whitney *U* test	<0.001
Fig. 6*F*		Nonparametric	Mann–Whitney *U* test	<0.05
Fig. 6*G*		Normally distributed	Unpaired *t* test	<0.001
Fig. 7B		Nonparametric	Mann–Whitney *U* test	<0.001
Extended Data Fig. 7-1*A*		Nonparametric	Wilcoxon matched-pairs signed-rank test	<0.05
Extended Data Fig. 7-1*B*		Nonparametric	Wilcoxon matched-pairs signed-rank test	<0.05
Fig. 8*C*		Nonparametric	Kruskal–Wallis test	<0.05 <0.001
Fig. 8*D*		Nonparametric	Kruskal–Wallis test	<0.01 <0.001
Fig. 8*E*		Nonparametric	Kruskal–Wallis test	<0.01 <0.001
Fig. 8*F*		Normally distributed	One-way ANOVA, Games–Howell *post hoc* test	<0.05 <0.001
Fig. 8*G*		Nonparametric	Kruskal–Wallis test	<0.05 <0.001

### Organotypic slices

All experimental procedures were conducted in compliance with the institutional guidelines of The University of Queensland Animal Ethics Committee. Wistar rat pups were provided by UQBR at postnatal day (P)4. Pups were euthanized by decapitation, and the hippocampus was removed. 400-µm slices were then taken of the hippocampus and placed onto sections of nitrocellulose membrane ∼5 mm^2^, which were suspended on a Millipore 50-nm net well in a six-well plate, containing 1 ml of culture medium. Culture medium was composed of 100-ml neurobasal medium, 50 ml HBSS, 50 ml heat inactivated horse serum, 2 ml D-glucose to make a total of 200 ml, and pH was set to 7.2. Following plating, hippocampal slices were maintained at 35°C in 5% carbon dioxide (CO_2_). Each well was treated with anti-mitotics (0.5 μm uridine, 0.5 μm ARA-C, 0.5 μm 5-Fluoro-deoxy-uridine) at 3 d *in vitro* (DIV) to inhibit glial growth. Net wells containing slices were then transferred to fresh plates and medium at 4 DIV to end treatment. Following this, medium changes were undertaken every 2–3 d by replacement of 500-µl medium from each well.

At 5 DIV for GFP and 8 DIV for Gas7 experiments, hippocampal slices were biolistically transfected with plasmids. This involved the coating of 1.5 µm Au particles with spermidine and 25 µg plasmid DNA. These particles were then deposited onto silicone tubing, which was cut and inserted into a Bio-Rad Gene Gun. Particles were then propelled into each well of hippocampal slices by He pressurized at 180 PSI.

Transfection success was assessed by fluorescence microscopy (Zeiss Discovery V8 Stereoscope) at 10 DIV for GFP and at 12–13 DIV for Gas7 experiments. Neurons showing visible expression of fluorescent proteins were selected for two-photon imaging.

At 10–13 DIV, neurons were imaged with a two-photon laser-scanning microscope (Bruker Ultima Investigator) using a 60×, 0.9 numerical aperture objective (Olympus) using a Ti:Sapphire laser (Mai Tai Deep See Laser, Newport-Spectra Physics) at 920 nm with a imaging intensity of 20mW, measured in objective back aperture. Green and red fluorescence were captured with dual close-proximity photomultiplier GaAsP detectors (Hamamatsu Model H10770) using emission filters (et525/70m-2p and et595/50m-2p) and a t565lpxr dichroic beam splitter for simultaneous viewing and acquisition from both detectors. Scan control and image acquisition was performed by Prairie View software. Either apical or basal dendritic segments were imaged. Image stacks consisted of sections (e.g., 40 × 40 µm) taken in 0.5-µm steps. The resolution of the imaging system (point spread function) was of x/y ∼0.6 µm and z ∼3.5 µm.

**Figure 1.**
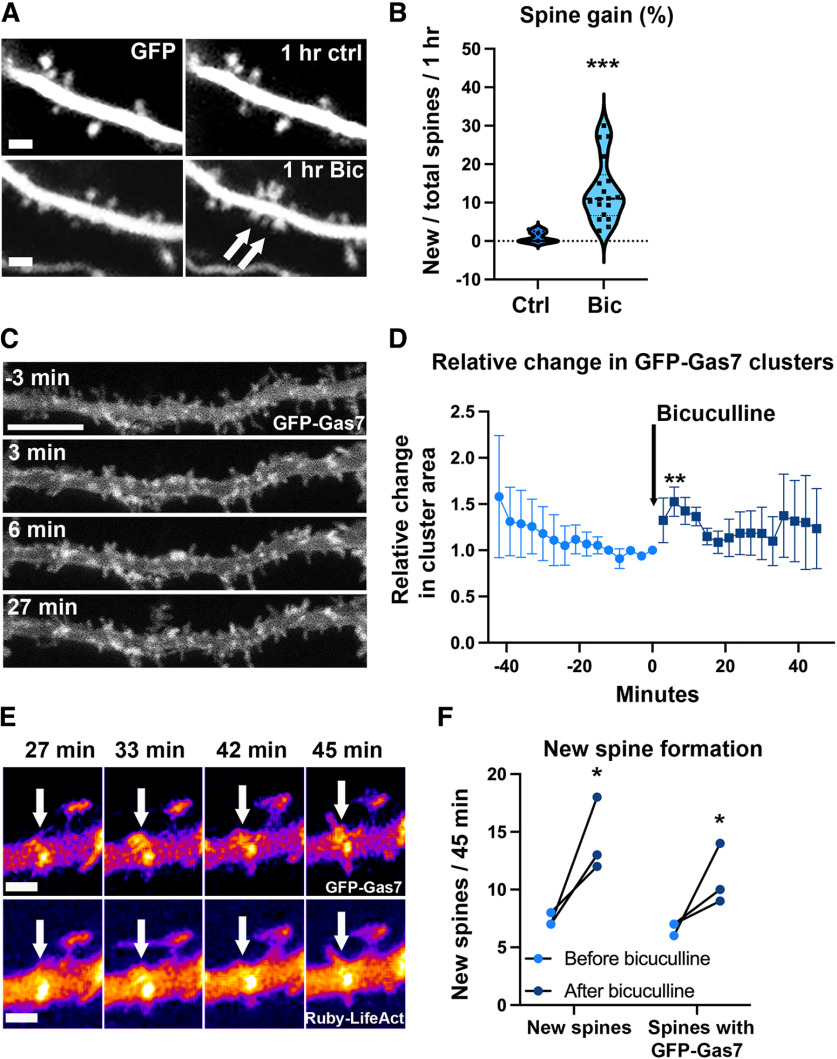
Bicuculline treatment-induced neuron activation increases spine formation and Gas7 clustering in organotypic slices. ***A***, Time frames for the dendrite of GFP-expressing hippocampal pyramidal neuron in DIV10 organotypic slice. Bicuculline-treated slices were treated for 1 h with 30 μm bicuculline. Newly formed spines after bicuculline treatment are shown by the arrows. Scale bar, 1 µm. ***B***, Quantification of the percentage of spine gain in control versus the bicuculline-treated neurons (pooled data from apical and basal dendrites). The new spines/total spines/1 h for control was 1.0 ± 0.63% compared with that of the bicuculline-treated neurons, 14.0 ± 2.4%. ***p < 0.001 as determined by Mann–Whitney *U* test *N*_(control)_ = 5 and *N*_(Bicuculline)_ = 12. Each cell is from different, independent slice. Violin plot presents the median and interquartile range (25th and 75th percentiles). ***C***, Time frames showing a segment of GFP-Gas7-transfected neuron before and after 30 μm bicuculline treatment. Scale bar, 5 µm. See also Movie 1 and Extended Data Figure 1-1. ***D***, Quantification of changes in the relative values for total area of GFP-Gas7 clusters before and after bicuculline treatment. *N* = 4 cells, the relative values for total area of GFP-Gas7 clusters increased significantly after bicuculline addition based on Wilcoxon paired test, **p < 0.01. Each cell is from different, independent slice from different animal. ***E***, Time frames of a dendritic segment expressing GFP-Gas7 and Ruby-LifeAct followed after bicuculline treatment. Black-and-white images are pseudocoloured based on intensity (Fire, Fiji). Arrows point to GFP-Gas7-enriched proto-protrusion where filopodium protrudes out. F-actin is slightly enriched in proto-protrusion. Scale bar, 1 µm. See also Movie 2. ***F***, New spines with or without preceding GFP-Gas7 cluster were counted before and after bicuculline treatment, both 45 min. Both the total number of new spines and the number of new spines with a preceding closely located GFP-Gas7 cluster were significantly increased after adding bicuculline based on Mann–Whitney *U* test, *p < 0.05. On average 85% of new spines had preceding GFP-Gas7.

During imaging, hippocampal slices were maintained in artificial CSF (aCSF; 127 mm NaCl, 2.5 mm KCl, 25 mm NaHCO_3_, 1.25 mm NaH_2_PO_4_, H_2_O, 2 mm CaCl_2_, 1 mm MgCl_2_, 25 mm d-glucose), circulated by a peristaltic pump. Following a baseline image at *t* = 0, the slices was perfused with aCSF containing 30 μm bicuculline (Tocris; treatment group) or continued in unadulterated aCSF (controls) and a second image was taken at *t* = 1 h for experiments shown in Figure 1*A*,*B*. Instead, in experiments presented in Figure 1*C–F*, we followed changes with time lapse imaging. A new stack started every 3 min.

10.1523/ENEURO.0344-22.2023.f1-1 Extended Data Figure 1-1 Extended reporting of cluster analysis of bicuculline-treated GFP-Gas7-expressing pyramidal neurons supporting Figure 1*C*,*D*. ***A***, Overall image intensity of the analyzed videos. Frames are acquired every 3 min and there are 15 frames before and after 30 µm bicuculline treatment. Overall intensity decreases in one cell, otherwise intensities are relatively stable. All analyses of the figure are from same dataset containing four GFP-Gas7-expressing pyramidal neurons each from independent organotypic slice. ***B***, Relative GFP-Gas7 fluorescence intensity in clusters and noncluster areas. GFP-Gas7 fluorescence intensity increases in all analyzed cells in cluster areas and it decreases in noncluster areas after adding bicuculline. Intensities are relative intensities so that intensity in clusters before bicuculline is set to 1. ***C***, Relative number of clusters slowly decreases during imaging. Addition of bicuculline did not show any change to gradual decrease. The relative number of clusters is calculated by dividing each of the original number of clusters by the average number of clusters obtained from the last three frames before adding bicuculline. ***D***, Average size of clusters before and after bicuculline treatment in µm^2^. Before is average of the last three frames before bicuculline treatment. After is highest average value in first 10 min after bicuculline treatment. Thus, this is the maximum change in cluster size. On average, cluster size doubles. Download Figure 1-1, TIF file.

### Analyses of organotypic slice experiments

For Figure 1*A*,*B*experiment, the total amount of spines and new spines were counted after a 1-h bicuculline treatment. The number of new spines was divided by the total spine number and this was given as % of new spines. Figure 1*E*,*F* experiment was analyzed similarly to Figure 1*A*,*B*, but as this was a time-lapse video, new spines were counted frame by frame and a new spine was marked to a frame when it appeared the first time. Simultaneously, new Gas7 clusters were marked to frames when they appeared the first time. As the number of new spines were low in each frame, 15 frames before and after bicuculline were pooled together. For Figure 1*C*,*D*, Gas7 or actin clustering was analyzed for each frame with a cluster analysis (see cluster analysis). We analyzed different parameters for clusters and the total area of clusters was decided to be representative parameter which was then used in figures. The brightness threshold value was individually set for each cell, so that the most of the visible clusters were included in the analysis. Then the cluster area was normalized so that the average of three last frames before the bicuculline treatment was set to be 1. All analyses were done from maximum projections of two-photon 3D stacks.

### Cluster analysis

From the original 3D image stack data files, the maximum intensity projection tiff images were created by using in Fiji software the option “Image -> Stacks -> Z project,” selecting all slices and using the projection type “Max Intensity.” This operation was done separately for each image channel of the original 3D image stack data file. To prevent background noise or crossing dendrites to be unintentionally included in the analysis, a region of interest (ROI) was manually defined for the neural image with a specific separate mask image. To define the ROI for a neural image, one-pixel-wide white curves on black background were manually drawn along the desired dendrite’s branches in Gimp software. These white curves were saved as a “skeleton curve image.” The width of these skeleton curves was then widened 95 pixels (5 µm) to the both sides from the curve [using the option “Select -> Grow… (Grow selection)” in Gimp software], thus getting 191-pixel-wide “observation paths” that were saved as a black-and-white mask image (white color indicating all the pixels to be included in the further analysis of the maximum intensity projection tiff image). The length of the region of interest (ROI) for the desired dendrite was computed from the “skeleton curve image” by using in Fiji software the option “Analyze -> Skeleton -> Analyze Skeleton (2D/3D).”

Motivated by the previous research and open source algorithm components (Hagler et al., 2006; Holmes and Huber, 2022), we developed and created a new cluster analysis method and programmed a new R language script named “Neural image brightness cluster analysis script” (Lahti, 2022b) to identify and analyze the emergence of brightness clusters in the given neural images (input images). The script relies on, among others, R language libraries magrittr, tidyverse, imager, BiocManager, locfit, magick, spatstat, EBImage, and ggplot2. The script takes as inputs the maximum intensity projection tiff images and their corresponding manually defined mask images. The script computes and outputs visualizations and exact numeric result files describing pixel regions that the script has identified in the input image file matching the conditions defined by the script’s adjustable parameter values about the minimum brightness threshold value (for example, 0.4). These numeric result files describe the number of identified brightness regions per image and the area of each of these brightness regions. To support identification of brightness clusters based on agglomerating separate but relatively closely positioned bright pixels, the script enables using supplementary smoothed versions of input image files to identify brightness regions. These supplementary smoothed versions of input image files can be generated with the script´s functionality that implements Gaussian blurring with desired adjustable parameter values of the size of “the brush” of the Gaussian kernel and the value of the σ for “the brush” of the Gaussian kernel. The full script and its related protocol and further details are described in the manuscript (Lahti, 2022a).

“Neural image brightness cluster analysis script” is also openly available at the following GitHub repository (https://github.com/laurilahti/neural-image-brightness-cluster-analysis-script). This script can be used on the public online computing environment of the Binder Project that is a nonprofit project built, led and supported by an open community (https://mybinder.org). To start the running of this script online you can access the following web address: https://mybinder.org/v2/gh/laurilahti/neural-image-brightness-cluster-analysis-script/HEAD?urlpath=rstudio. When running the script “neural-image-brightness-cluster-analysis-script-developed-by-lauri-lahti.R” for the first time, the initialization and installation phases may take a long time (for example, 30 min or more) before the actual analysis results become generated. However, after that the subsequent new running of the script generates the actual analysis results much more quickly. Usage guidance for the script can be found at the GitHub repository web site (https://github.com/laurilahti/neural-image-brightness-cluster-analysis-script). The Binder Project online environment is designed to address carefully data privacy and security when running a script online in this environment (https://mybinder.readthedocs.io/en/latest/about/user-guidelines.html#security-and-privacy). Anyway, if the user wants to analyze sensitive data with the script, it is recommended to be done so that the script is running only in the user’s own local computer.

When using the script to identify brightness clusters in neural images (input images), the correct brightness threshold value needs to be set manually based on the shown visualizations. In an optimal situation, with this brightness threshold value the script identifies a similar selection of clusters as a person would identify independently without the script. However, achieving this was sometimes difficult, especially when analyzing input images captured from time-lapse videos in which the cluster intensity, total brightness, or cluster/diffuse protein ratio changed after drug treatment.

Setting the correct brightness threshold value depended largely on the brightness distribution of the image so that a wide dynamic range could be exploited well but in some cases the brightness threshold value had to be set so that only a narrow dynamic range could be exploited to prevent the script to identify incorrectly bright diffuse stainings as clusters. When analyzing images of fixed cells in different experiments, the same brightness threshold value was set for all images that belonged to the same experiment. As an exception, in Figure 6, N-WASP was relatively diffuse without WT GFP-Gas7 expression. Similarly, mCherry-actin, expressed together with WA, was diffuse in most neurons. For these neurons, the script did not manage to identify clearly distinct clusters. Therefore, for N-WASP analyses, threshold was visually selected cell by cell. WA and mCherry-actin expressing cells were analyzed first manually based on a visual inspection, and if there were no clusters in cell, these cells were given a value of 0.

### Neuronal cultures and transfections

Neuronal culture preparation has been described previously (Bertling et al., 2012). Animals were handled according to the Finnish laws and ethics under the EU directive 2010/63/EU (licenses: ESAVI-4943-04.10.07-2016, ESAVI/7404/2021, and GMO 3/S/12). In brief, the brains from Wistar rat fetuses of embryonic day 17 were collected. The meninges were removed to isolate and dissect the hippocampi. The hippocampal cells were dissociated in 0.05% papain, mechanically triturated, and suspended in Ca^2+^ and Mg^2+^-free HBBS medium with DNase I (20 U/ml, Sigma-Aldrich), sodium pyruvate (1 mm) and HEPES (10 mm, pH 7.2). The cells were then plated on 13 mm diameter glass coverslips coated with poly-L-lysine (0.01 mg/ml, Sigma-Aldrich). A total of 100,000 to 150,000 cells per 24-well plate well were cultured on 13-mm coverslips in Neurobasal medium (Invitrogen) supplemented with L-glutamine (Invitrogen), B-27 (Invitrogen), and primocin (InvivoGen). The neurons were cultured in humidified incubators at 37°C and 5% carbon dioxide (CO_2_), and the media was refreshed at regular intervals twice a week. Transient transfection was performed on DIV14 neurons using Lipofectamine 2000 (Invitrogen) as described previously (Hotulainen et al., 2009). The transfected neurons were fixed after 24 h of the transfection except for shRNA-transfected neurons that were fixed at 3 d after the transfection. Latrunculin B and PI3-kinase inhibitor LY294002 were purchased from Sigma-Aldrich.

### Immunofluorescence and antibodies

The neurons were fixed using 37°C 4% paraformaldehyde (PFA) for 20 min at room temperature and then washed three times with PBS. The neurons were then permeabilized using 0.2% Triton X-100 in PBS. Blocking was done for 30 min using 3% normal donkey serum and 0.5% bovine serum albumin (BSA) in PBS. Antibodies were diluted in blocking solution and coverslips were stained setting them upside down over a drop of antibody solution. The neurons were incubated in primary antibody for 1 h at room temperature and washed three times for 10 min with 0.2% BSA in PBS. The neurons were then incubated in the secondary antibody for 1 h and then washed three times for 10 min in a similar way. Subsequently, the coverslips with neurons were mounted on glass slides by using Shandon Immu-Mount (Thermo Fisher Scientific).

The mouse monoclonal anti-Gas7 antibody (sc-365385) was purchased from Santa Cruz Biotechnology and the mouse anti-myc (9E10) antibody was purchased from Thermo Fisher Scientific. The dilution of 1:200 was used for both primary antibodies. The anti-mouse Alexa-647 was purchased from Thermo Fisher Scientific and the dilution of 1:400 was used.

### Western blotting

C57Bl6 mice were anesthetized and transcardially perfused using 50-ml cold PBS. Cortex, hippocampus, and cerebellum from the right hemisphere were fast frozen in liquid nitrogen and stored at −80°C for western blotting. Tissue was then homogenized and lysed in the mixture of radio immunoprecipitation assay (RIPA) buffer [50 mm Tris–HCl pH 7.4, 150 mm NaCl, 1 mm EDTA, 0.25% sodium deoxycholate and 1% NP-40, 1% SDS and phosphatase and protease inhibitor cocktail (Roche)]. Bicinchoninic acid (BCA) protein assay (Thermo Fisher Scientific) was used to estimate the protein concentration. For each sample, 35 µg protein was run on 10% SDS-PAGE gel. The manufacturer’s protocol was followed to transfer the protein from the gel to the polyvinylidene difluoride (PVDF) membrane; 5% milk in TBS-0.1% Tween 20 (TBS-T) was used for blocking the membrane for 1 h at the room temperature. The membrane was then incubated overnight at 4°C in primary antibody against Gas7 (mouse, 1:1000, Santa Cruz Biotechnology) diluted in 5% milk in TBS-T. Next, the membrane was washed 3 times 10 min with TBS-T and incubated in secondary antibody (anti-mouse, 1:2500, Invitrogen) at the room temperature for 1 h. After three times 10-min wash with TBS-T, enhanced chemiluminescence (ECL) reagent (Thermo Fisher Scientific) was used to develop the membrane and detect the specific protein bands. The membrane was stripped using stripping buffer (2% glycine) and the same protocol was repeated with mouse anti-actin AC-15 antibody (1:5000; Sigma-Aldrich) to detect the total actin. Gas7 levels were quantified against total protein using Image Lab software.

### Imaging

Confocal images were obtained using either Zeiss LSM780 or LSM880 inverted confocal microscope. The fixed samples were imaged at room temperature and the live cell imaging was performed in a chamber where the temperature and CO_2_ levels can be adjusted. The live cells were imaged in culture media at 37°C and 5% CO_2_. The 63× 1.4 NA oil immersion objective was used for imaging. Z-stack of 20–30 optical sections was obtained for each neuron. The step size was set to 0.2 μm. The laser power and gain settings were adjusted to maximize the signal-to-noise ratio. The Fiji software was used to process the image files obtained from the imaging (Schindelin et al., 2012, 2015).

### Counting and characterizing new protrusions from time-lapse videos

The Fiji software was used to quantify the number of new protrusions. First, the video with maximum intensity projections was obtained from the 3D video data. Each time frame of the video was analyzed compared with the previous time frame for the formation of new protrusions. For Gas7-overexpressing neurons, protrusions initiated from the Gas7 clusters were marked. Moreover, the presence or absence of membrane curvature before the protrusion formation was also noted. The “straight line” tool was used to measure the length of the analyzed region of the dendrite. Finally, the number of new protrusions/10 µm/min, percentage of new protrusions originating from Gas7 clusters, as well as the percentage of new protrusions from Gas7 clusters that preceded with membrane curvature were quantified. The new protrusion analysis for LY294002 was also done in a similar way to quantify and compare the number of new protrusions/10 µm/min and percentage of new protrusions initiating from Gas7 clusters before and after the LY294002 treatment.

### Dendritic spine morphology and density analysis

NeuronStudio was used for the analysis of dendritic spine morphology and density. NeuronStudio is a software package designed for three-dimensional detection of dendritic spines from fluorescence microscopy images (Rodriguez et al., 2008). It also allows the classification of spines into different types based on their morphology. The Fiji software was used to obtain the tiff files with the z-stacks of 20–30 optical sections. The images had the voxel size of 0.066 × 0.066 × 0.2 μm and the EGFP or the mCherry channel was used for analyzing the dendritic spine morphology and density.

After modeling of the dendrite surface, protrusions with a minimum volume of five voxels (0.020 μm^3^), length between 0.1 and 5 μm, and a maximal width of 3 μm were retained as spines. Following the default settings of the program and the empirical classification rule defined by Rodriguez et al. (2008), spines with a minimum head diameter of 0.35 μm and a minimum head versus neck ratio of 1.1 were classified as mushroom spines. Nonmushroom spines with a minimum volume of 10 voxels (0.040 μm^3^) were classified as stubby spines. All other spines were considered thin. The spines detected and modeled by the software were carefully verified and corrected manually. The structures wrongly labeled as spines were removed and the spines that were not detected by the software were added and classified. Measurements obtained by NeuronStudio were transferred to a spreadsheet application (MS Excel) for analysis.

### Protein localization analysis

The Fiji software was used for the protein localization analysis as described previously (Hlushchenko et al., 2018). The images with maximum intensity projections were obtained from the 3D image data. A circular region of interest (ROI) was selected in a spine head and an equal-sized ROI was selected in the adjacent dendrite. The intensity values obtained were normalized to the intensity distribution of mCherry in the same ROIs. Then, the ratio of average intensity of fluorescence was calculated. The process was repeated in at least 10 spines for each neuron and the median was taken as the representative value for the neuron.

### shRNA knock-down analysis

The Fiji software was used to quantify the effect of Gas7 shRNA knock-down. First, the images with maximum intensity projections were obtained from the 3D image data. The anti-Gas7 647 signal was used for the shRNA knock-down analysis. First, the cell body of the shRNA-transfected neuron was selected as a ROI and the intensity value was measured. Similarly, intensity values from the cell bodies of neighboring nontransfected neurons were obtained. Next, the relative Gas7 fluorescence intensity was calculated by comparing the intensity obtained from the transfected neuron to the mean intensity of neighboring nontransfected neurons. Finally, the mean values were normalized to the mean relative intensity value obtained from neurons expressing the scramble shRNA.

### Colocalization analysis

The colocalization analysis between mCherry-actin and GFP-Gas7 signals was conducted on the maximum intensity projections obtained from the 3D image data. The Z-stacks at the best focus for each channel were manually selected for the maximum intensity projection. An outline image defining the region of interest for the colocalization analysis was created using GIMP, which is an open-source drawing and annotation software (https://www.gimp.org/). The EzColocalization Plugin in the Fiji software was used for the colocalization analysis (Stauffer et al., 2018; Lahti, 2021; Micinski et al., 2022). Pearson’s correlation coefficient (PCC) value was calculated for each image to determine the degree of co-occurrence of two signals. PCC values range from −1, which reflects a strong anti-colocalization, to 1, which reflects strong colocalization. PCC value of 0 reflects that there is no correlation between the two signals.

### Statistical analyses

Statistical analyses were conducted using IBM SPSS Statistics software package (IBM Corp.). Statistical significance was determined using *t* test or Mann–Whitney *U* test when comparing two groups. The Wilcoxon paired test was used for the paired datasets. The ANOVA with appropriate *post hoc* tests or Kruskal–Wallis test was used when comparing more than two groups. Shapiro–Wilk test was performed to check the normality of the data and Levene’s test was used to determine the homogeneity of variance in the data. If the variances were equal, Bonferroni’s *post hoc* was used and in the case of unequal variances, Games–Howell *post hoc* was used. The summary of the statistical analyses is shown in Table 2.

### Data availability

The datasets generated during and/or analyzed during the current study are not yet publicly available but are available from the corresponding author on reasonable request.

## Results

### F-BAR domain containing Gas7 clusters on neuron activation

Hamilton et al. (2012) showed earlier that bicuculline treatment enhanced synaptic activity in organotypic slices and it resulted in a 69% increase in spine outgrowth at days *in vitro* (DIV)7–DIV12. We first confirmed these results using DIV10 organotypic slices (Fig. 1*A*,*B*). At DIV10, 30 μm bicuculline treatment significantly increased the gain of new spines, both at basal and apical dendrites (Fig. 1*A*,*B*; pooled data). At control conditions, only a few new spines appeared over 1 h (Fig. 1*A*,*B*). With bicuculline treatment, the rate of spine initiation significantly increased, resulting in on average 13 new spines per 100 existing spines after 1-h treatment. Next, we tested whether some BAR domain containing proteins respond to neuron activation by bicuculline. One of the tested proteins, Gas7, showed fast and repeatable response to bicuculline treatment. We routinely transfected organotypic slices at DIV8 and an optimal expression time window for Gas7 was 4–5 d, thus, DIV12–DIV13 were used for additional experiments. We first measured whether bicuculline treatment affected overall image intensities but image intensity was relatively stable in all analyzed cells (Extended Data Fig. 1-1*A*). We next analyzed manually whether GFP-Gas7 fluorescence intensity changed in bright GFP-Gas7 clusters or in the areas of no clusters. This analysis showed that GFP-Gas7 fluorescence intensity increased in clusters and decreased in noncluster areas (Extended Data Fig. 1-1*B*). It seemed that the diffuse cytoplasmic GFP-Gas7 was relocated next to plasma membrane to form bigger or brighter clusters. As manual analysis is vulnerable for personal biases, we developed a new R language script named “Neural image brightness cluster analysis script” (Lahti, 2022b) to analyze clusters. Our script identifies clusters in a neural image by identifying pixels that have a fluorescence intensity that is greater than a determined brightness threshold value. The script also takes into account how these pixels form clusters. The script reports the number of clusters, the fluorescence intensity in clusters and the area of clusters as well as visualizations to support manual verification (see Materials and Methods for further information and open access for using the script online). Analysis of bicuculline-treated neurons showed that number of clusters did not increase on bicuculline treatment compared with situation before treatment. In contrast, relative number of clusters slowly decreased throughout the whole imaging time and bicuculline treatment did not affect this decrease (Extended Data Fig. 1-1*C*). To have a good comparison before and after bicuculline treatment, we followed same cell 45 min before treatment and 45 min after adding bicuculline. The average size of clusters doubled shortly after adding bicuculline (Extended Data Fig. 1-1*D*). As the average number of clusters was decreasing and the average size of clusters was increasing, we decided that the best way to report results is to use total cluster areas. These analyses showed that total cluster area increased especially during first 12 min and the average increase was 1.5-fold at 6 min after adding bicuculline (Fig. 1*C*,*D*; Movie 1). As the effect was relatively fast, the most probable mechanism to increase GFP-Gas7 intensity in clusters seemed to be that diffuse GFP-Gas7 relocates to clusters making them bigger. In time-lapse videos, GFP-Gas7 clustering was observed before a new spine formation was initiated (Fig. 1*E*; Movie 2). Bicuculline treatment enhanced spine initiation, increasing both the total number of new spines and the number of new spines which had a closely located GFP-Gas7 cluster before spine initiation (Fig. 1*F*). On average 85% of new spines had preceding GFP-Gas7 cluster. These results suggest that neuronal activation by bicuculline enhances Gas7 clustering and Gas7 clustering promotes formation of new spines (Fig. 1*C–F*).

Movie 1. Time-lapse video showing a segment of GFP-Gas7 and tdTomato transfected pyramidal neuron in DIV12 organotypic slice. Upper panel shows segment before treatment and lower panel shows same dendrite after 30 μM bicuculline treatment. Left panel shows GFP-Gas7 channel in inverse color. Panel on right shows merged GFP-Gas7 (green) and tdTomato (red). Time frames of GFP-Gas7 channel are shown in Figure 1*C*. Frames are imaged every 3 minutes. Both before and after have 15 frames = 45 minutes. Frame rate in video is 8 frames / second. 10.1523/ENEURO.0344-22.2023.video.1

Movie 2. Time-lapse video showing a segment of GFP-Gas7 and Ruby-LifeAct transfected pyramidal neuron in DIV12 organotypic slice after 30 μM bicuculline treatment. Left upper panel shows GFP-Gas7 channel in inverse color. Panel below shows Ruby-LifeAct in inverse color. Right upper panel shows Gas7 in inverse Fire and below right is merged GFP-Gas7 (green) and Ruby-LifeAct (red). Arrows point to site where new filopodium appears. Time frames of video are shown in Figure 1*E*. Frames are imaged every 3 minutes. Video shows 10 frames = 30 minutes. Frame rate in video is 8 frames / second. 10.1523/ENEURO.0344-22.2023.video.2

Movie 3. Time-lapse video showing a segment of RFP-LifeAct and GFP-Gas7 transfected pyramidal neuron in DIV14 primary hippocampal culture. Upper panel shows segment before treatment and lower panel shows same dendrite after 5 μM Latrunculin B treatment. Left panel shows RFP-LifeAct channel in inverse color. Panel on right shows GFP-Gas7 in inverse color. Time frames of video are shown in Figure 5D. Frames are imaged every 3 minutes. Both before and after have 10 frames = 30 minutes. Frame rate in video is 8 frames / second. 10.1523/ENEURO.0344-22.2023.video.3

Movie 4. Time-lapse video showing a segment of Akt PH domain-mRFP and GFP-Gas7 transfected pyramidal neuron in DIV21 primary hippocampal culture. One frame is shown in Figure 7*C*. Left panel shows GFP-Gas7 channel and middle shows Akt PH-mRFP in inverse color. Right panel shows merged GFP-Gas7 (green) and Akt PH-mRFP (red). Arrows point to one typical Gas7-cluster where GFP-Gas7 and Akt PH-mRFP co-localize. Frames are imaged every 60 seconds and video has 15 frames = 15 minutes. Frame rate in video is 8 frames / second. 10.1523/ENEURO.0344-22.2023.video.4

### Gas7 localizes to spine initiation sites in primary hippocampal neurons and Gas7 overexpression increases the spine density

**Figure 2.**
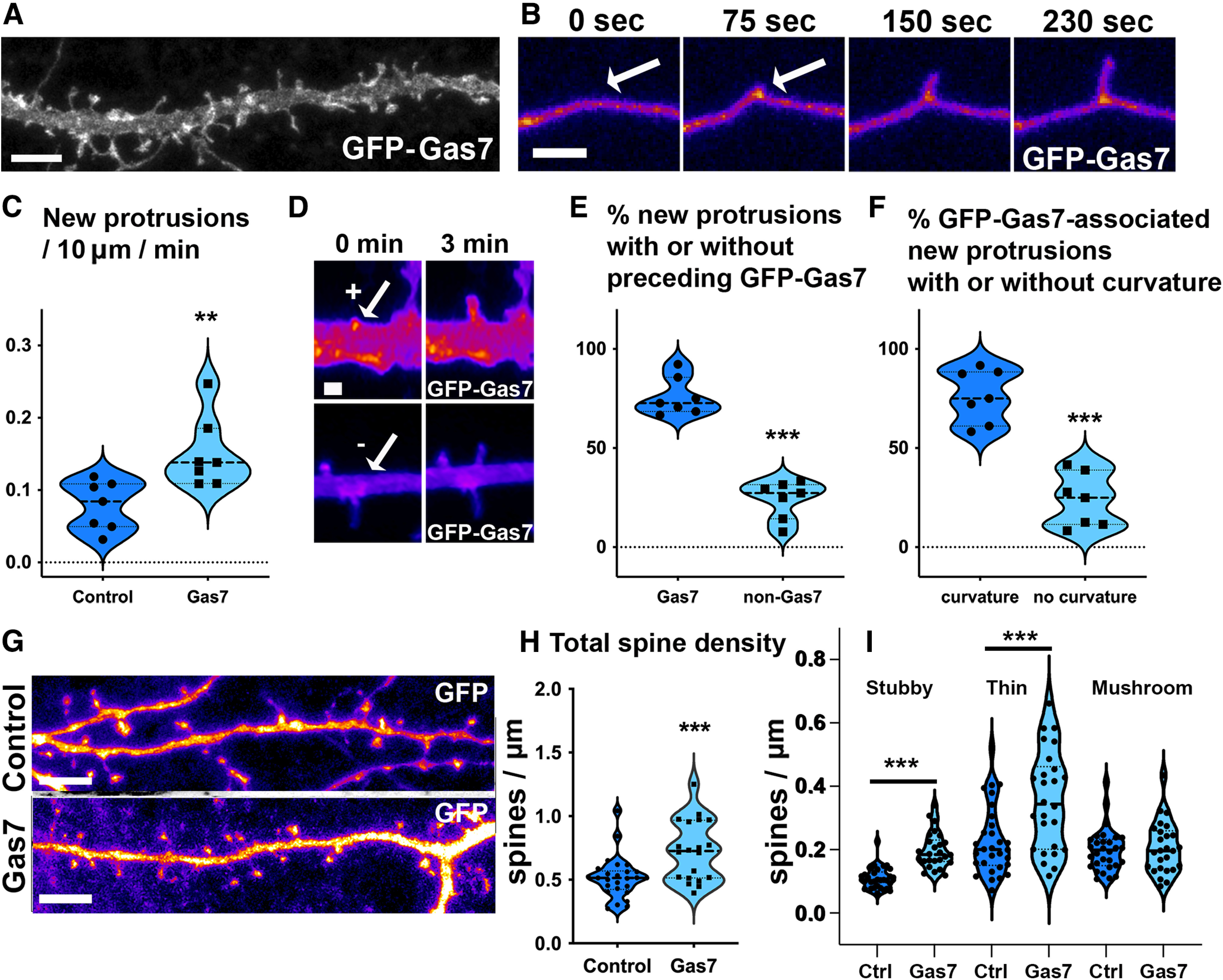
Gas7 localizes to clusters on dendrites before new filopodia formation and its overexpression increases spine density in the primary hippocampal neurons. ***A***, Representative dendritic segment of a hippocampal DIV15 neuron transfected with GFP-Gas7. Scale bar, 5 μm. ***B***, Time frames of a segment of hippocampal neuron transfected with GFP-Gas7. New filopodium emerge from GFP-Gas7 cluster on dendritic membrane as shown by the arrow. Scale bar, 2 μm. Pseudocolored with intensity-based Fire. ***C***, Quantification of the rate of formation of new protrusions in control versus the Gas7-overexpressing neurons. The average number of new protrusions/10 µm/1 min for control was 0.078 ± 0.012 compared with that of the Gas7-overexpressing neurons, 0.150 ± 0.018. **p < 0.01 as determined by unpaired *t* test (control: *n* = 7 videos, 64 spines; Gas7: *n* = 7 videos, 143 spines). ***D***, Representative examples of GFP-Gas7 positive (+) and negative (−) new filopodia. Scale bar, 1 μm. Pseudocolored with intensity-based Fire. ***E***, Quantification of the percentage of new protrusions initiating with and without a GFP-Gas7 cluster at the base of the newly formed protrusion in Gas7-overexpressing neurons. The average percentage of new protrusions initiating from GFP-Gas7 clusters was 75.9 ± 3.6 compared with the new protrusions not initiating from GFP-Gas7 clusters, 24.1 ± 3.6. ***p < 0.001 as determined by unpaired *t* test (*n* = 7 videos, 143 spines). ***F***, Quantification of the percentage of Gas7-initiated new protrusions that preceded with membrane curvature versus the ones preceded without the membrane curvature. The average percentage of Gas7-initiated new protrusions that preceded with membrane curvature was 76.3 ± 5.1 compared with the Gas7-initiated new protrusions that did not precede with membrane curvature 23.7 ± 5.1. ***p < 0.001 as determined by unpaired *t* test (*n* = 7 videos, 106 clusters). See also Extended Data Figure 2-1. ***G.*** Representative images showing dendritic segments of neurons overexpressing GFP along with mCherry or mCherry-Gas7. GFP was used for quantification shown in ***H***, ***I***. Scale bar, 5 μm. Pseudocolored with intensity-based Fire. ***H***, Quantification of total dendritic spine density calculated as number of spines per 1 µm of a dendrite. Spine density for control was 0.534 ± 0.032 and for mCherry-Gas7-expressing cells was 0.758 ± 0.049. ***p < 0.001 as determined by unpaired *t* test (control: *n* = 30 neurons; Gas7: *n* = 26 neurons). Data were pooled from five independent experiments. ***I***, Quantification of dendritic spine density of different types of spines calculated as number of stubby, thin, or mushroom spines per 1 µm of a dendrite. For control, stubby spine density was 0.109 ± 0.006, thin spine density was 0.226 ± 0.019 and mushroom spine density was 0.198 ± 0.012 whereas for mCherry-Gas7-expressing neurons, stubby spine density was 0.197 ± 0.011, thin spine density was 0.355 ± 0.030 and mushroom spine density was 0.205 ± 0.015. ***p < 0.001 as determined by unpaired *t* test (control: *n* = 30 neurons; Gas7: *n* = 26 neurons). Data were pooled from five independent experiments. Data are represented as mean ± SEM. Violin plots present the median and interquartile range (25th and 75th percentiles).

We further characterized Gas7 in primary hippocampal neurons and observed that GFP-Gas7 localized to small clusters on the plasma membrane (Fig. 2*A*), similarly to neurons in organotypic slices. To test whether these clusters are indeed spine initiation sites, as was seen for MIM (Saarikangas et al., 2015), and for Gas7 in organotypic slices, we examined the spatiotemporal localization of GFP-Gas7 during spine formation (Fig. 2*B*). GFP-Gas7 clustering was associated with membrane bending (75 s, arrow) and followed by filopodium outgrowth (150 s; Fig. 2*B*). Overexpression of Gas7 nearly doubled the spine initiation rate from 0.08 new protrusions/10 µm dendrite length per minute to 0.15 (Fig. 2*C*). We next visually determined how often GFP-Gas7 cluster was preceding a new spine outgrowth (Fig. 2*D*). In GFP-Gas7-expressing cells, three of four new spines were initiated from GFP-Gas7-clusters and one fourth of spines were initiated elsewhere (Fig. 2*E*). We showed earlier that MIM curved the plasma membrane and induced a proto-protrusions facilitating spine initiation (Saarikangas et al., 2015). Although Gas7 is not directly curving membrane *in vitro* (Hanawa-Suetsugu et al., 2019), we analyzed whether GFP-Gas7-clusters preceding new spines were curved or not curved (flat). We found out that GFP-Gas7 clusters associated with different curvatures: either broad and relatively flat curvature, resembling small lamellipodium, or with more curved and smaller protrusion, resembling a small stubby spine (Extended Data Fig. 2-1). Visual analysis revealed that three of four GFP-Gas7-clusters leading to new spine initiation were curved and one fourth were not curved (Fig. 2*F*). Thus, although Gas7 F-BAR is not effectively curving the membrane *in vitro* (Hanawa-Suetsugu et al., 2019), the appearance of GFP-Gas7 on membrane is associated with membrane curvature. These results indicate that Gas7 is a novel spine initiation factor, which contribute to formation of proto-protrusions on the plasma membrane thus facilitating initiation of a new spine.

10.1523/ENEURO.0344-22.2023.f2-1 Extended Data Figure 2-1 Extended data supporting Figure 2*F*. GFP-Gas7-associated membrane curvatures preceding new filopodia formation. Time frames of a segment of hippocampal neurons transfected with GFP-Gas7. New filopodia often emerged from GFP-Gas7 clusters with different membrane curvatures. Curvature is visualized with drawn line on left. Time frames are 3 min apart from each other. Arrows point to areas were new filopodia form. Scale bar, 1 μm. Pseudocolored with intensity-based Fire. Download Figure 2-1, TIF file.

To assess the effect of Gas7 overexpression on spine density, the neurons were co-transfected with GFP together with mCherry or mCherry-Gas7. GFP was used for spine analysis (Fig. 2*G*). We used the NeuronStudio software for the dendritic spine density and morphology analysis (Rodriguez et al., 2008; Fig. 2*H*,*I*). Comparison of the number of dendritic spines in neurons expressing GFP either with mCherry or mCherry-Gas7 revealed that Gas7 overexpression significantly increased the dendritic spine density (Fig. 2*H*). Gas7 overexpression mainly increased stubby and thin spines but had no effect on mushroom spines (Fig. 2*I*). These results showed that GFP-Gas7 clusters are associated with spine initiation and overexpression of GFP-Gas7 positively affects spine density.

### Gas7 is highly expressed in the developing and adult mouse brain

Gas7 has different splicing isoforms, which are differently expressed in different cell types. In addition to the F-BAR domain, Gas7b possesses a WW domain, Gas7cb and Gas7c possess a WW and an SH3 domain, while Gas7d contains only the F-BAR domain. Among these, Gas7b with WW- and F-BAR-domains (molecular weight ∼48 kDa) seems to be the main neuronal isoform (Ju et al., 1998; Tseng et al., 2015; Zhang et al., 2016).

**Figure 3.**
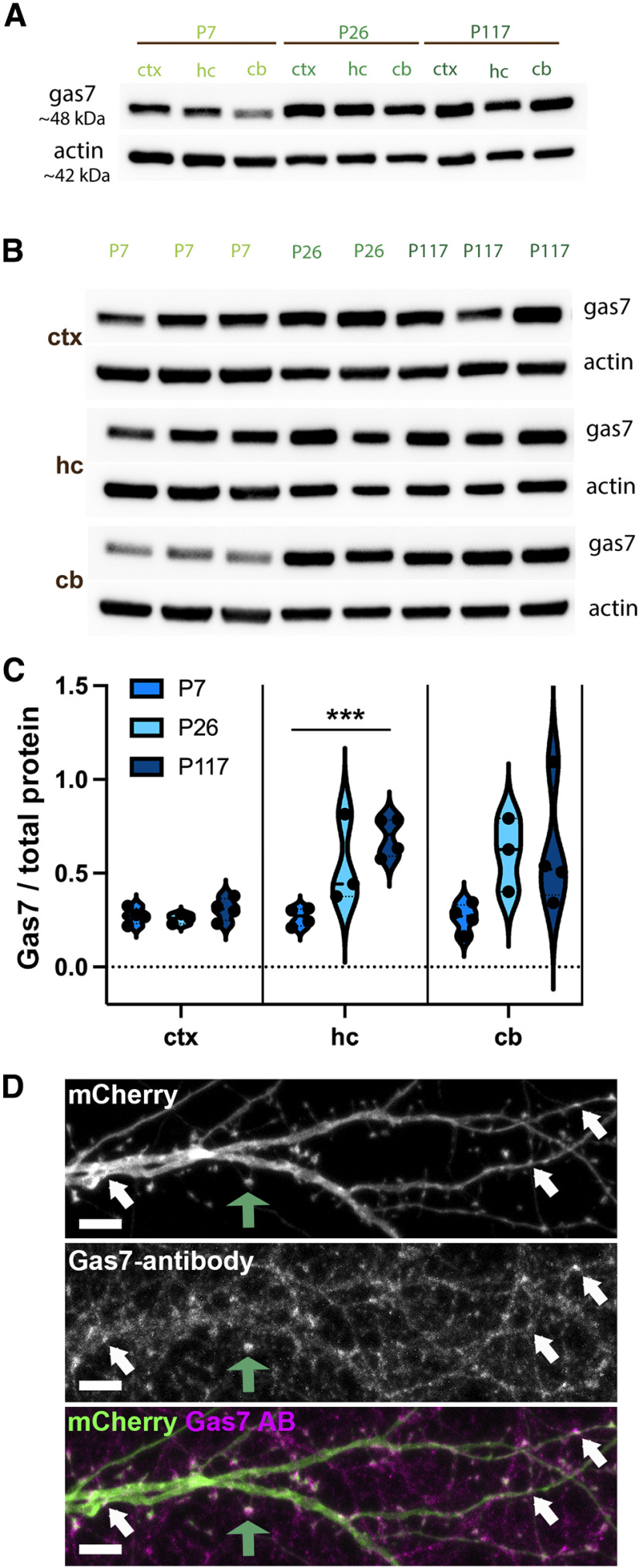
Gas7 is widely expressed in the developing and adult brain. ***A***, Tissues obtained from cortex (ctx), hippocampus (hc), and cerebellum (cb) of P7, P26, and P117 mice were run on SDS-PAGE gel and blotted against Gas7 and actin. Gas7 protein runs around ∼48 kDa and actin runs ∼42 kDa. ***B***, Cortex, hippocampus and cerebellum tissue samples of three P7, two P26, and three P117 mice were run on SDS-PAGE gel and blotted against Gas7 and actin. ***C***, Relative Gas7 expression level was quantified against total protein levels. In hippocampus (hc), the Gas7 expression increases significantly when P7 samples (3 mice, 4 Western blottings) were compared with P117 samples (3 mice, 4 Western blottings). ***p < 0.001, unpaired *t* test. Violin plot presents the median and interquartile range (25th and 75th percentiles). ***D***, Anti-Gas7 immunostaining of 15 DIV hippocampal neurons transfected with mCherry. Endogenous Gas7 is enriched in small clusters on dendrites as shown by the white arrows as well as in spine heads (one spine head is highlighted with green arrow). Scale bars, 5 µm.

To examine the expression of Gas7 in the developing and adult brain, we performed Western blot analysis in the brain tissues obtained from P7, P26, and P117 mice (Fig. 3*A–C*). Western blot analysis showed that Gas7 is expressed in both young and adult mouse brains (Fig. 3*A*,*B*). Quantification of Gas7 levels relative to total protein showed that Gas7 was less expressed at P7 compared with later time points in hippocampus (hc) and cerebellum (cb). In cortex (ctx), the Gas7 expression stayed at low level also at later time points (Fig. 3*C*).

To analyze the localization of endogenous protein in primary hippocampal neurons, we immunostained mCherry-transfected cells with anti-Gas7 antibodies. These experiments revealed that endogenous Gas7 localization is comparable to GFP-Gas7 localization shown in Figure 2, concentrating on small clusters on the dendrites and in spine heads (Fig. 3*D*, highlighted with arrows). These results show that Gas7 is expressed in tested brain areas throughout the mouse life and that endogenous protein exhibits similar localization as overexpressed GFP-tagged constructs.

### Gas7 knock-down decreases dendritic spine density in the primary hippocampal neurons

**Figure 4.**
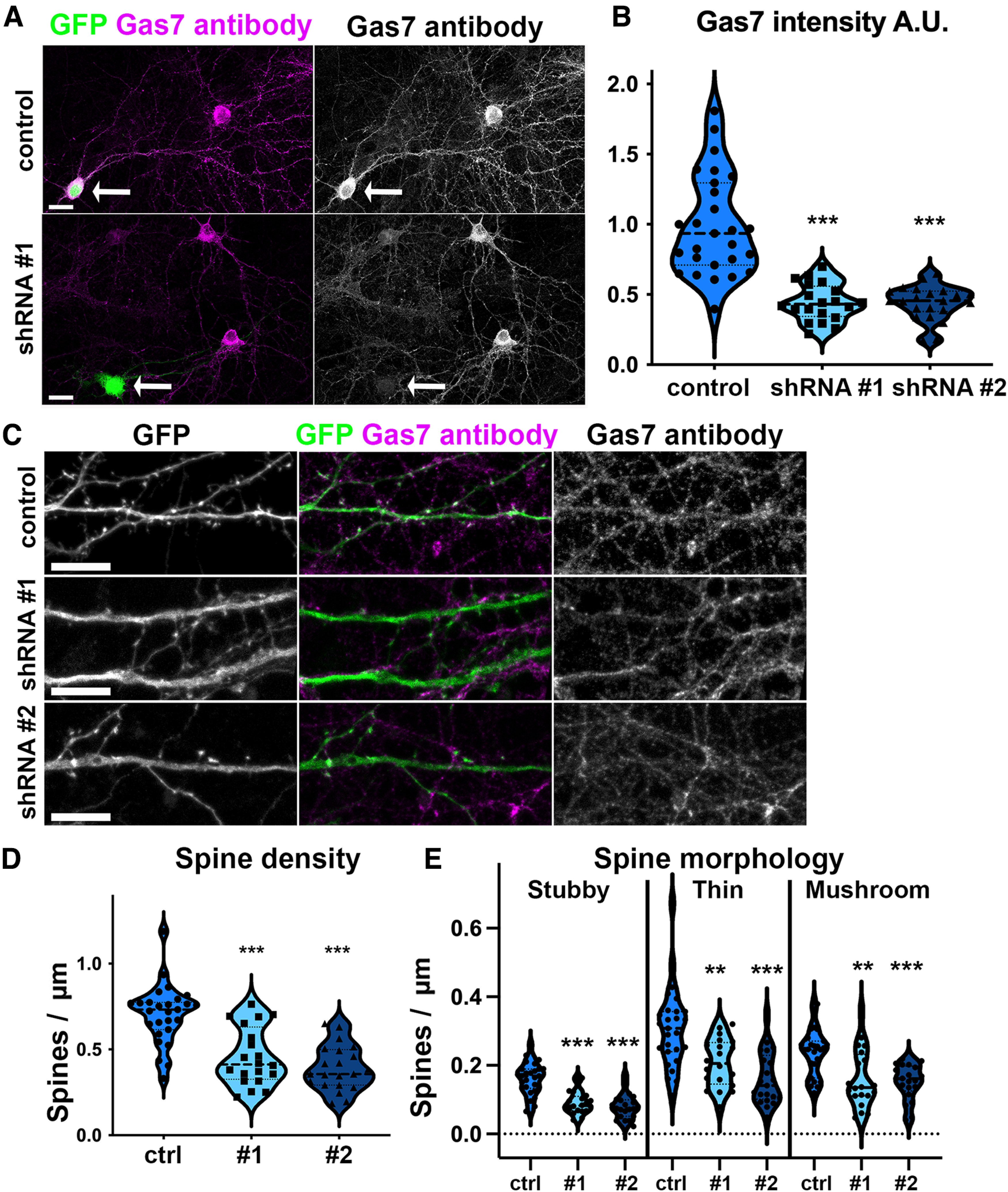
Gas7 knock-down decreases spine density in primary hippocampal neurons. ***A***, Representative images of the 17 DIV hippocampal neurons expressing GFP and scrambled shRNA or Gas7-targeted shRNA. 14 DIV hippocampal neurons were transfected with GFP and either scrambled shRNA or one of the two anti-Gas7 shRNAs (somas of transfected cells are highlighted with arrows). The neurons were fixed after 3 d followed by an immunostaining with anti-Gas7 antibody. Scale bars, 20 µm. ***B***, Quantification of Gas7 intensity in neurons expressing Gas7 shRNA. The intensity obtained for shRNA expressing neurons was then normalized with the scrambled shRNA expressing neurons. Data are pooled from four experiments and represented as mean ± SEM, ***p < 0.001 as determined by one-way ANOVA with Games–Howell *post hoc* test. Gas7 intensity for shRNA #1 was 0.442 ± 0.031 and shRNA #2 was 0.441 ± 0.027 (control: *n* = 24 neurons; shRNA #1: *n* = 19 neurons; shRNA #2: *n* = 21 neurons). ***C***, Representative dendritic segments of hippocampal neurons expressing GFP and scrambled shRNA or Gas7 shRNA #1 or Gas7 shRNA #2. GFP channel was used for analyzing dendritic spine density (***D***) and morphology (***E***). Immunostaining with anti-Gas7 was used to confirm the reduction of endogenous Gas7 expression. Scale bars, 10 µm. ***D***, Quantification of dendritic spine density for neurons expressing scrambled shRNA, Gas7 shRNA #1, and Gas7 shRNA #2. ***p < 0.001 as determined by one-way ANOVA with Bonferroni’s *post hoc* test. Spine density for control = 0.705 ± 0.032, shRNA #1 = 0.458 ± 0.037 and shRNA #2 = 0.392 ± 0.028 (control: *n* = 27 neurons; shRNA #1: *n* = 20 neurons; shRNA #2: *n* = 21 neurons). ***E***, Quantification of dendritic spine density of different types of spines for neurons expressing scrambled shRNA, Gas7 shRNA #1, and Gas7 shRNA #2. The dendritic spine density for different spine types was calculated as number of stubby, thin, or mushroom spines per 1 µm of a dendrite. Data are pooled from 4 experiments and represented as mean ± SEM, **p < 0.01 and ***p < 0.001 as determined by one-way ANOVA with Bonferroni’s *post hoc* test. For control, stubby spine density was 0.161 ± 0.008, thin spine density was 0.307 ± 0.022 and mushroom spine density was 0.236 ± 0.013. For shRNA #1, stubby spine density was 0.090 ± 0.007, thin spine density was 0.207 ± 0.015 and mushroom spine density was 0.160 ± 0.018. For shRNA #2, stubby spine density was 0.080 ± 0.007, thin spine density was 0.160 ± 0.016 and mushroom spine density was 0.151 ± 0.011(control: *n* = 27 neurons; shRNA #1: *n* = 20 neurons; shRNA #2: *n* = 21 neurons). Violin plots present the median and interquartile range (25th and 75th percentiles). See also Extended Data Figure 4-1.

We next tested whether knock-down of Gas7 affects dendritic spine density. For that, we used two different shRNA constructs targeting the Gas7 mRNA. We transfected the primary hippocampal neurons with GFP and either scrambled shRNA or one of the two anti-Gas7 shRNAs (Fig. 4*A*). Figure 4*A* shows cells transfected with control shRNA (control) and shRNA targeted to Gas7 (shRNA #1, arrows highlight somas of transfected cells). The fluorescence intensity of anti-Gas7 antibody staining in the soma of Gas7 shRNA-transfected cell is very low compared with neighboring nontransfected cells demonstrating that shRNA treatment has significantly decreased the expression of Gas7 in this cell. The comparison of anti-Gas7 antibody staining intensity in somas of transfected neurons to nontransfected neighboring neurons revealed that the expression of anti-Gas7 shRNA reduced the level of Gas7 to less than half from its normal expression level (Fig. 4*A*,*B*). For spine density and morphology analysis, we acquired images with higher magnification (Fig. 4*C*). GFP intensity was used to analyze the dendritic spine density and morphology. Neurons expressing shRNAs targeted to Gas7 showed significantly reduced total dendritic spine density compared with the neurons expressing scrambled shRNA (Fig. 4*D*). The shRNA-mediated knock-down of Gas7 led to the significant reduction in all spine morphologies (Fig. 4*E*). We further tested whether spine phenotype associated with knock-down of endogenous Gas7 can be rescued with human GFP-Gas7 construct which mismatches with four and two bases with Gas7 shRNA target sequences #1 and #2, respectively (Extended Data Fig. 4-1). Expression of human GFP-Gas7 construct rescued decreased Gas7 expression and Gas7 knock-down spine phenotype (Extended Data Fig. 4-1). Taken together, reduction in Gas7 expression level decreases the spine density and spine density can be rescued by shRNA resistant GFP-Gas7 overexpression.

10.1523/ENEURO.0344-22.2023.f4-1 Extended Data Figure 4-1 Extended data supporting Figure 4. Human GFP-Gas7 overexpression rescues Gas7 knock-down induced spine phenotype. ***A***, Representative dendritic segments of hippocampal neurons expressing mCherry together with scrambled shRNA (ctrl), Gas7 shRNA #1 (#1 and R #1), or Gas7 shRNA #2 (#2 and R #2). ctrl, #1 and #2 are also transfected with GFP. In R #1 and R #2, knock-down of endogenous Gas7 is rescued by overexpressing human GFP-Gas7, which is resistant to used shRNAs. All cells are stained with anti-Gas7 antibody. mCherry channel was used for analyzing dendritic spine density and morphology (B). mCherry on right is shown as intensity-based pseudocoloring (Fire). All scale bars, 10 µm. On left, is shown anti-Gas7 antibody staining in magenta and either GFP or GFP-Gas7 in green. In the middle, anti-Gas7 antibody staining is shown in black and white to see different expression levels in transfected cell somas compared to neighboring nontransfected cells. In control cells transfected with scrambled shRNA, Gas7 staining is at similar level compared to nontransfected cells. In Gas7 shRNA-transfected, but not rescued, cells (#1 and #2), Gas7 expression is lower than in neighboring cells. In cells rescued with GFP-Gas7 expression (R #1 and R #2), Gas7 level is higher than in neighboring cells and transfected cells are easy to distinguish from nontransfected cells. White arrows point to somas of transfected cell somas. mCherry images on right (higher zoom in of cells shown on left) show that Gas7 shRNA-transfected cells have less spines compared to controls or rescued cells. ***B***, Quantification of total dendritic spine density for neurons expressing scrambled shRNA (ctrl), Gas7 shRNA #1 (#1), and Rescued shRNA #1 (R #1). The total spine density was ctrl = 0.68 ± 0.071, #1 = 0.38 ± 0.036, R #1 = 1.02 ± 0.062. Ctrl: *n* = 16 neurons; #1: *n* = 15 neurons; R #1 16 neurons. Data is pooled from three experiments and represented as mean ± SEM. **p < 0.01 and ***p < 0.001 as determined by one-way ANOVA with Bonferroni’s *post hoc* test. Violin plots present the median and interquartile range (25th and 75th percentiles). ***C***, Quantification of dendritic spine density of different types of spines for neurons expressing scrambled shRNA (ctrl), Gas7 shRNA #1 (#1), and Rescued shRNA #1 (R #1). Stubby spine densities were ctrl 0.16 ± 0.016, #1 = 0.089 ± 0.010, R #1 = 0.24 ± 0.019. Thin spine densities were ctrl 0.29 ± 0.032, #1 = 0.16 ± 0.017, R #1 = 0.46 ± 0.033. Mushroom spine densities were ctrl 0.23 ± 0.036, #1 = 0.14 ± 0.027, R #1 = 0.32 ± 0.027. Data is pooled from three experiments and represented as mean ± SEM. *p < 0.05 and **p < 0.01 as determined by one-way ANOVA or Kruskal–Wallis test. Violin plots present the median and interquartile range (25th and 75th percentiles). ***D***, Quantification of total dendritic spine density for neurons expressing scrambled shRNA (ctrl), Gas7 shRNA #2 (#2), and Rescued shRNA #2 (R #2). The total spine density was ctrl = 0.68 ± 0.071, #2 = 0.38 ± 0.033, R #2 = 1.07 ± 0.066. Ctrl: *n* = 16 neurons; #2: *n* = 15 neurons; R #2 16 neurons. Data is pooled from three experiments and represented as mean ± SEM. **p < 0.01 and ***p < 0.001 as determined by one-way ANOVA with Games–Howell *post hoc* test. Violin plots present the median and interquartile range (25th and 75th percentiles). ***E***, Quantification of dendritic spine density of different types of spines for neurons expressing scrambled shRNA (ctrl), Gas7 shRNA #2 (#2), and Rescued shRNA #2 (R #2). Stubby spine densities were ctrl 0.16 ± 0.016, #2 = 0.092 ± 0.0081, R #2 = 0.25 ± 0.014. Thin spine densities were ctrl 0.29 ± 0.032, #2 = 0.13 ± 0.014, R #2 = 0.50 ± 0.044. Mushroom spine densities were ctrl 0.23 ± 0.036, #2 = 0.16 ± 0.023, R #2 = 0.32 ± 0.026. Data is pooled from three experiments and represented as mean ± SEM. *p < 0.05, **p < 0.01, and ***p < 0.001 as determined by one-way ANOVA or Kruskal–Wallis test. Violin plots present the median and interquartile range (25th and 75th percentiles). Download Figure 4-1, TIF file.

### Gas7 co-localizes with F-actin at the dendritic spine initiation sites

**Figure 5.**
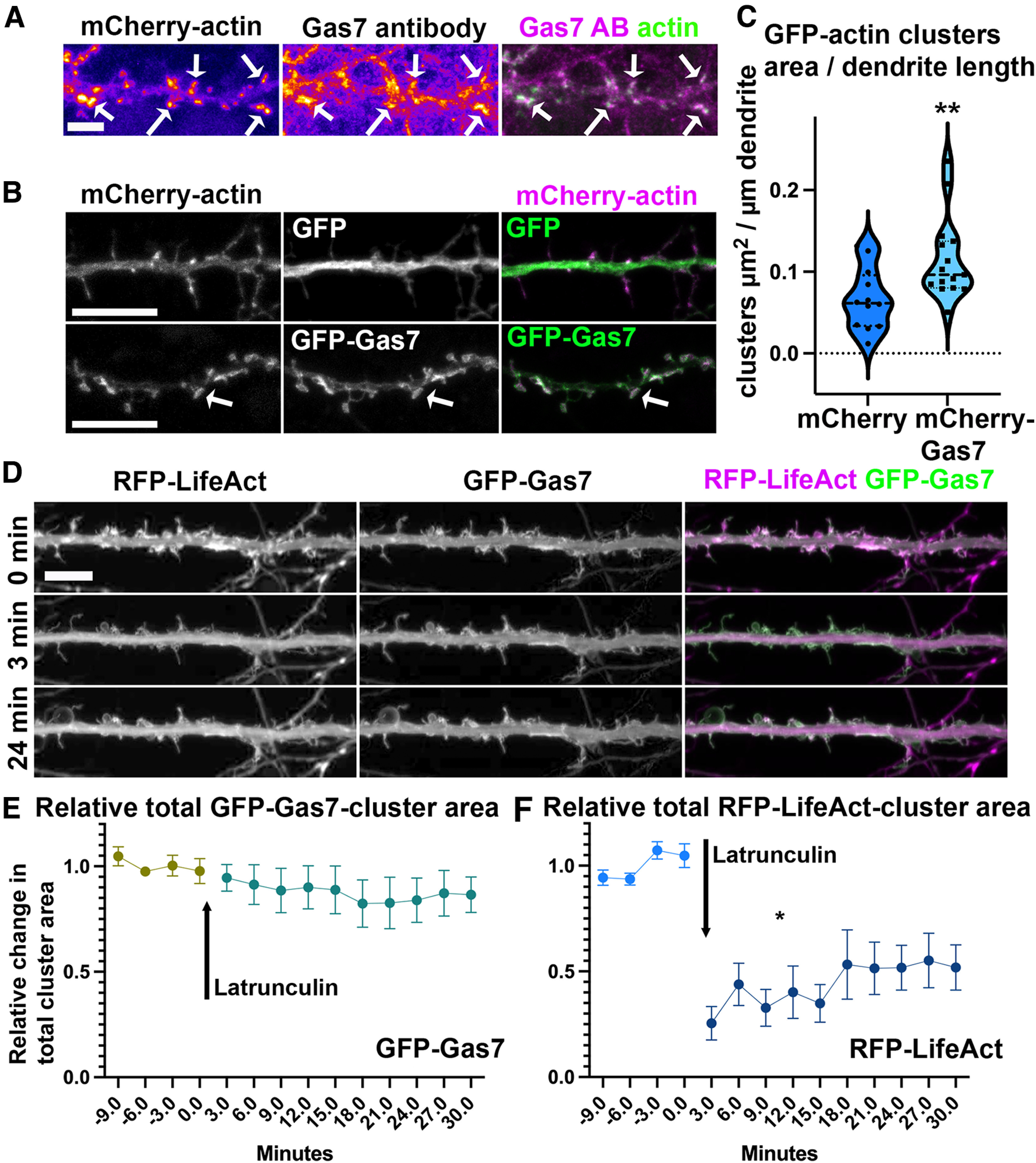
Gas7 and actin localize to same clusters and Gas7-overexpression increases clustering of actin. ***A***, Dendritic segment of a primary hippocampal neuron transfected with mCherry -actin and immunostained with anti-Gas7 antibody. Endogenous Gas7 localized to mCherry-actin clusters as shown by the arrows. Scale bar, 5 µm. ***B***, Upper panel shows a dendritic segment of a primary hippocampal neuron expressing mCherry-actin and GFP whereas the lower panel shows a dendritic segment of a neuron expressing mCherry-actin and GFP-Gas7. Scale bars, 10 µm. ***C***, Quantification of GFP-actin clustering in primary hippocampal neurons expressing mCherry or mCherry-Gas7. Data are pooled from three experiments. **p < 0.01 as determined by Mann–Whitney *U* test (GFP-actin + mCherry: *n* = 12 neurons; GFP-actin + mCherry-Gas7: *n* = 15 neurons). For GFP-actin + mCherry, cluster area (µm^2^) per dendrite length (µm) was 0.066 ± 0.011 and for GFP-actin + mCherry-Gas7, cluster area per length was 0.115 ± 0.013 (mean ± SEM). Violin plot presents the median and interquartile range (25th and 75th percentiles). ***D***, Time frames of a segment of primary hippocampal neuron transfected with RFP-LifeAct and GFP-Gas7 before and after 5 μm latrunculin B treatment. While the RFP-LifeAct cluster localization is immediately changed to diffuse localization after latrunculin B treatment (first time point after latrunculin B is 3 min), GFP-Gas7 clusters remain largely unaffected. See also Movie 3. ***E***, GFP-Gas7 cluster analysis shows that total GFP-Gas7 cluster area is slowly decreasing on 5 μm latrunculin B treatment. Six cells were analyzed from 3 independent experiments. Brightness threshold value for cluster analysis was set based on general intensity. Plot shows mean ± SEM of relative values. Relative values are calculated based on average of cluster area in 4 frames before adding latrunculin B. Change in intensity is thus shown as a relative change to original values. Wilcoxon paired test shows that change in GFP-Gas7 clusters is not significant *p* = 0.44. ***F***, Same analysis as in ***E*** for RFP-LifeAct. RFP-LifeAct clusters are lost immediately after addition of latrunculin B but clusters recover during the follow-up. Wilcoxon paired test shows that change in RFP-LifeAct clusters is significant *p < 0.05.

We showed earlier that actin polymerization was required for filopodia growth after initial formation of a proto-protrusion by MIM/Mtss1 (Saarikangas et al., 2015). Also, the SrGAP3-induced spine formation involves regulation of the actin cytoskeleton (Khanal and Hotulainen, 2021). To elucidate whether the actin cytoskeleton has a role in Gas7-induced spine initiation, we compared the localization of Gas7 and actin and tested the effect of Gas7 overexpression on the actin cytoskeleton and the effect of actin depolymerization on GFP-Gas7 localization. Endogenous Gas7 and expressed mCherry-actin localize to same clusters, both on dendritic shaft and in spines (Fig. 5*A*, arrows). However, closer examination showed that localization of endogenous Gas7 and mCherry-actin was not always exactly the same, but it seemed that actin forms “a core” and Gas7 is around this core. The GFP-Gas7 co-localized with mCherry-actin more exactly (Fig. 5*B*, arrows). We next analyzed the GFP-actin clustering in mCherry-Gas7-expressing cells and compared the clustering to control neurons expressing mCherry. This analysis showed that Gas7 overexpression enhances the clustering of actin (Fig. 5*C*). As it seemed that Gas7 and actin co-assemble, we tested whether actin polymerization is required for the maintenance of Gas7 localization and clustering. For this, we treated GFP-Gas7 and RFP-LifeAct expressing neurons with latrunculin B which sequesters actin monomers and thus inhibits the polymerization of actin filaments resulting in F-actin depolymerization (Fig. 5*D*; Movie 3). Upon latrunculin B treatment, clustered RFP-LifeAct became diffuse indicating that filamentous actin was dissolved at first 3 min. GFP-Gas7 clusters were maintained although there seemed to be also more diffuse GFP-Gas7 than before treatment (Fig. 5*D*). Cluster analysis showed that GFP-Gas7 total cluster area was slowly decreasing throughout imaging (Fig. 5*E*). In contrast, RFP-LifeAct total cluster area decreased significantly after latrunculin B treatment. Decrease in total cluster area was reversible as RFP-LifeAct marked F-actin clusters were slowly recovering during 30 min follow-up imaging (Fig. 5*F*). In summary, F-actin was not necessary for maintaining Gas7 clusters. Together these results show that Gas7 and actin filaments co-localize in clusters, Gas7 increases actin clustering and F-actin is not required for the maintenance of Gas7 clusters.

**Figure 6.**
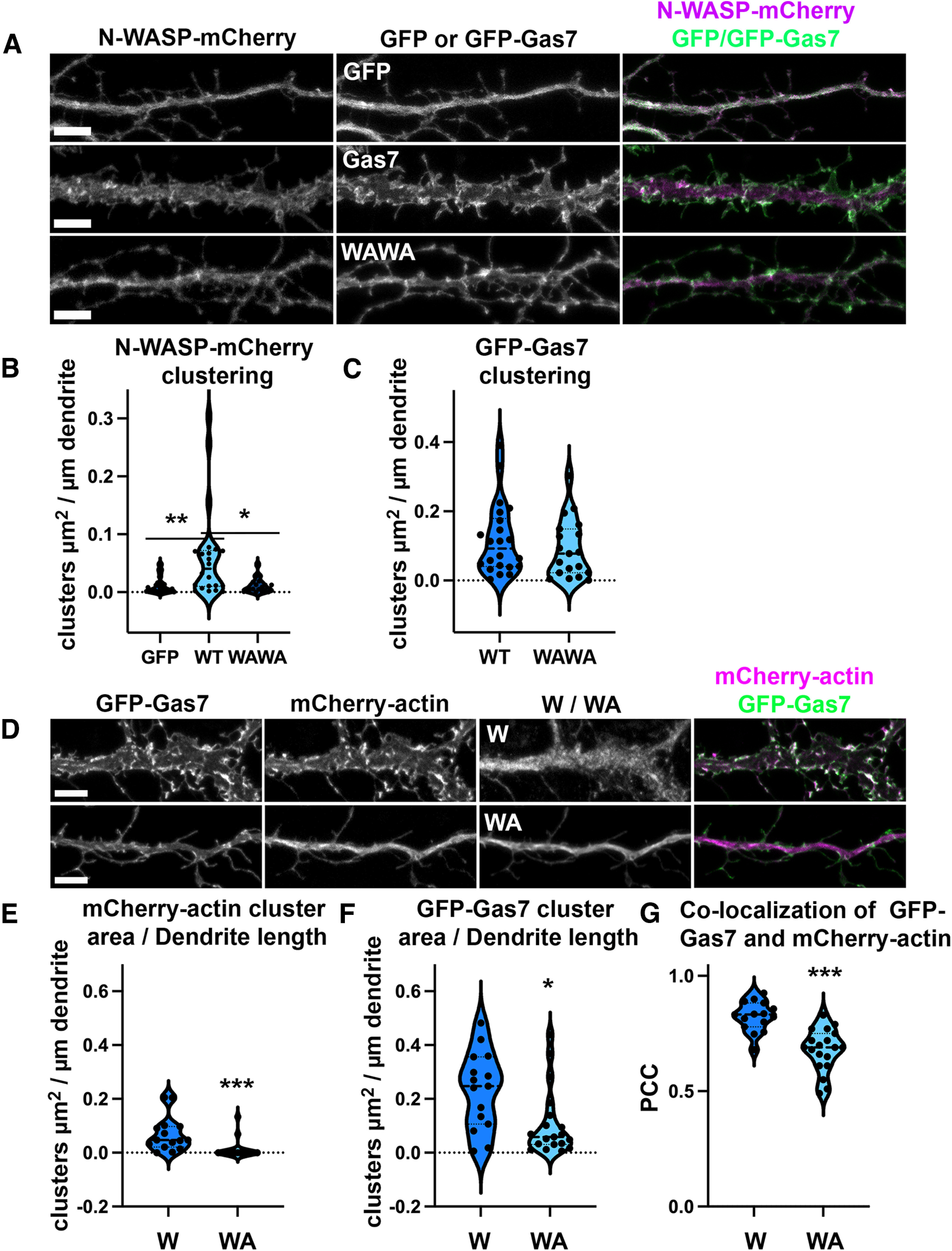
Gas7 mediates N-WASP localization and Arp2/3 complex is required for Gas7-induced actin clustering. ***A***, The upper panel shows a dendritic segment of a primary hippocampal neuron expressing N-WASP-mCherry and GFP. The middle panel includes a dendritic segment of a neuron expressing N-WASP-mCherry and GFP-Gas7 and the lower panel shows a dendritic segment of a neuron transfected with N-WASP-mCherry and mutated GFP-Gas7 in which both tryptophans of WW domain are mutated to alanine (WAWA). Scale bars, 5 µm. ***B***, Quantification of N-WASP-mCherry clustering in primary hippocampal neurons transfected with GFP (GFP), wild-type GFP-Gas7 (WT) and WW domain mutated GFP-Gas7 (WAWA) together with N-WASP-mCherry. Data are pooled from three experiments. Violin plot presents the median and interquartile range (25th and 75th percentiles). There is a significant change in N-WASP-mCherry clustering in GFP versus GFP-Gas7-expressing cells and in GFP-Gas7 WT versus GFP-Gas7 WAWA mutation expressing cells. *p < 0.05 and **p < 0.01 as determined by Kruskal–Wallis test. GFP: *n* = 29 neurons, WT: *n* = 22 neurons; WAWA: *n* = 19 neurons. N-WASP-mCherry cluster area (µm^2^) per dendrite length (µm) was 0.012 ± 0.003 for GFP, 0.062 ± 0.017 for WT GFP-Gas7 and 0.011 ± 0.003 for WAWA GFP-Gas7 (mean ± SEM). ***C***, Quantification of GFP-Gas7 clustering in primary hippocampal neurons transfected with wild-type GFP-Gas7 (WT) and WW domain mutated GFP-Gas7 (WAWA) with N-WASP-mCherry. Data are pooled from three experiments. Violin plot presents the median and interquartile range (25th and 75th percentiles). There is not a significant change in Gas7 clustering with WAWA mutation (WT: *n* = 22 neurons; WAWA: *n* = 19 neurons). GFP-Gas7 cluster area (µm^2^) per dendrite length (µm) was 0.117 ± 0.022 for WT GFP-Gas7 and 0.091 ± 0.019 for WAWA GFP-Gas7 (mean ± SEM). ***D***, The panel shows the representative image of a dendritic segment of a neuron transfected with GFP-Gas7, mCherry-actin and Scar W-myc or Scar-WA-myc. Myc-tag was immunostained with anti-myc secondary antibody linked to Alexa-647. Scale bars, 5 µm. ***E***, Quantification of mCherry-actin clustering in primary hippocampal neurons transfected with GFP-Gas7, mCherry-actin and Scar W-myc or Scar WA-myc. Data are pooled from three experiments and represented as mean ± SEM, ***p < 0.001 as determined by Mann–Whitney *U* test (W: *n* = 15 neurons; WA: *n* = 19 neurons). mCherry-actin cluster area (µm^2^) per dendrite length (µm) was 0.068 ± 0.017 for Scar W-myc-expressing neurons and 0.010 ± 0.008 for Scar WA-myc-expressing neurons. Violin plot presents the median and interquartile range (25th and 75th percentiles). ***F***, Quantification of GFP-Gas7 clustering in primary hippocampal neurons transfected with GFP-Gas7, mCherry-actin, and Scar W-myc or Scar WA-myc. Data are pooled from three experiments and represented as mean ± SEM, *p < 0.05 as determined by Mann–Whitney *U* test (W: *n* = 15 neurons; WA: *n* = 19 neurons). GFP-Gas7 cluster area (µm^2^) per dendrite length (µm) was 0.232 ± 0.037 for Scar W-myc-expressing neurons and 0.110 ± 0.029 for Scar WA-myc-expressing neurons. Violin plot presents the median and interquartile range (25th and 75th percentiles). ***G***, Quantification of the colocalization between GFP-Gas7 and mCherry-actin in Scar W-myc and Scar WA-myc-expressing neurons. To determine the co-occurrence of the two signals, Pearson’s correlation coefficient (PCC) value was calculated for each image. PCC values range from −1, which reflects a strong anti-colocalization, to 1, which reflects strong colocalization. PCC value of 0 reflects that there is no correlation between the two signals. Data are pooled from three experiments and represented as mean ± SEM, ***p < 0.001 determined by unpaired *t* test (W: *n* = 15 neurons; WA: *n* = 19 neurons). PCC value for Scar W-myc-expressing neurons was 0.823 ± 0.016 and PCC value for Scar WA-myc-expressing neurons was 0.68 ± 0.021. Violin plot presents the median and interquartile range (25th and 75th percentiles).

### Gas7 regulates the localization of N-WASP

The interaction between Gas7 and N-WASP through the WW domain in Gas7 has been previously reported (You and Lin-Chao, 2010). N-WASP is an actin nucleation promoting factor (NPFs) and an important regulator of actin dynamics (Rohatgi et al., 1999; Yamaguchi et al., 2000). N-WASP is also involved in MIM/Mtss1-driven spine initiation (Saarikangas et al., 2015). To test whether Gas7 modulates the localization of N-WASP, we compared the localization of N-WASP-mCherry in primary hippocampal neurons with and without GFP-Gas7 (Fig. 6*A*). When co-expressed with GFP alone, N-WASP-mCherry localized diffusively throughout the dendrite and spines (Fig. 6*A*). The co-expression of N-WASP-mCherry with GFP-Gas7 led to the clustering and accumulation of N-WASP-mCherry to the regions where GFP-Gas7 was clustering (Fig. 6*A*). Cluster analysis showed that when N-WASP-mCherry was co-expressed with GFP-Gas7, the area of N-WASP-mCherry clusters/dendrite length was significantly increased (Fig. 6*B*). This suggests that Gas7 regulates the localization of N-WASP in neurons. Consistent with this, the expression of GFP-Gas7 in which both tryptophans of WW domain were mutated to alanine (W19 and W41) reduced the Gas7-induced clustering of N-WASP-mCherry (Fig. 6*A*,*B*, WAWA). WW domain tryptophan mutations did not significantly affect GFP-Gas7 clustering (Fig. 6*C*) indicating that observed effect on N-WASP-mCherry clustering depends on Gas7 binding to N-WASP but not clustering of Gas7 itself.

### Arp2/3 complex activity is required for the Gas7-induced actin clustering

N-WASP binds and activates the Arp2/3 complex, a key actin nucleator (Rohatgi et al., 1999). Thus, we next tested whether the activity of Arp2/3 complex is necessary for Gas7-mediated actin clustering. For that, we used the Scar WA-myc construct. WA is a conserved sequence domain in proteins of the WASP family that binds to the Arp2/3 complex. Overexpression of this domain in cells disrupts the localization of the Arp2/3 complex, thus inhibiting its normal functionality (Machesky and Insall, 1998). Scar W-myc construct, which does not bind to the Arp2/3 complex, was used as a negative control (Machesky and Insall, 1998; Fig. 6*D*). The analysis of the neurons expressing GFP-Gas7 and actin-mCherry with either Scar WA or Scar W revealed that disrupting the Arp2/3 complex localization significantly reduced clustering of mCherry-actin (Fig. 6*D*,*E*). This indicates that the Arp2/3 complex is required for the Gas7-mediated actin clustering which also indicates that clustering is probably because of increased actin polymerization. Inhibition of Arp2/3 complex-induced actin polymerization decreased GFP-Gas7 clustering indicating that Gas7 can assemble in clusters without Arp2/3 complex but Arp2/3 complex-driven actin polymerization facilitates the growth of clusters (Fig. 6*F*). This is in line with our previous finding that latrunculin treatment did not abolish GFP-Gas7 localization indicating that Gas7 can assemble to clusters without actin polymerization (Fig. 5*D*,*E*). We further analyzed co-localization of GFP-Gas7 and mCherry-actin. This analysis showed that co-localization of GFP-Gas7 and mCherry-actin was reduced when Arp2/3 complex localization was disrupted (Fig. 6*G*).

### PI3K activity is required for Gas7 clustering in primary hippocampal neurons

**Figure 7.**
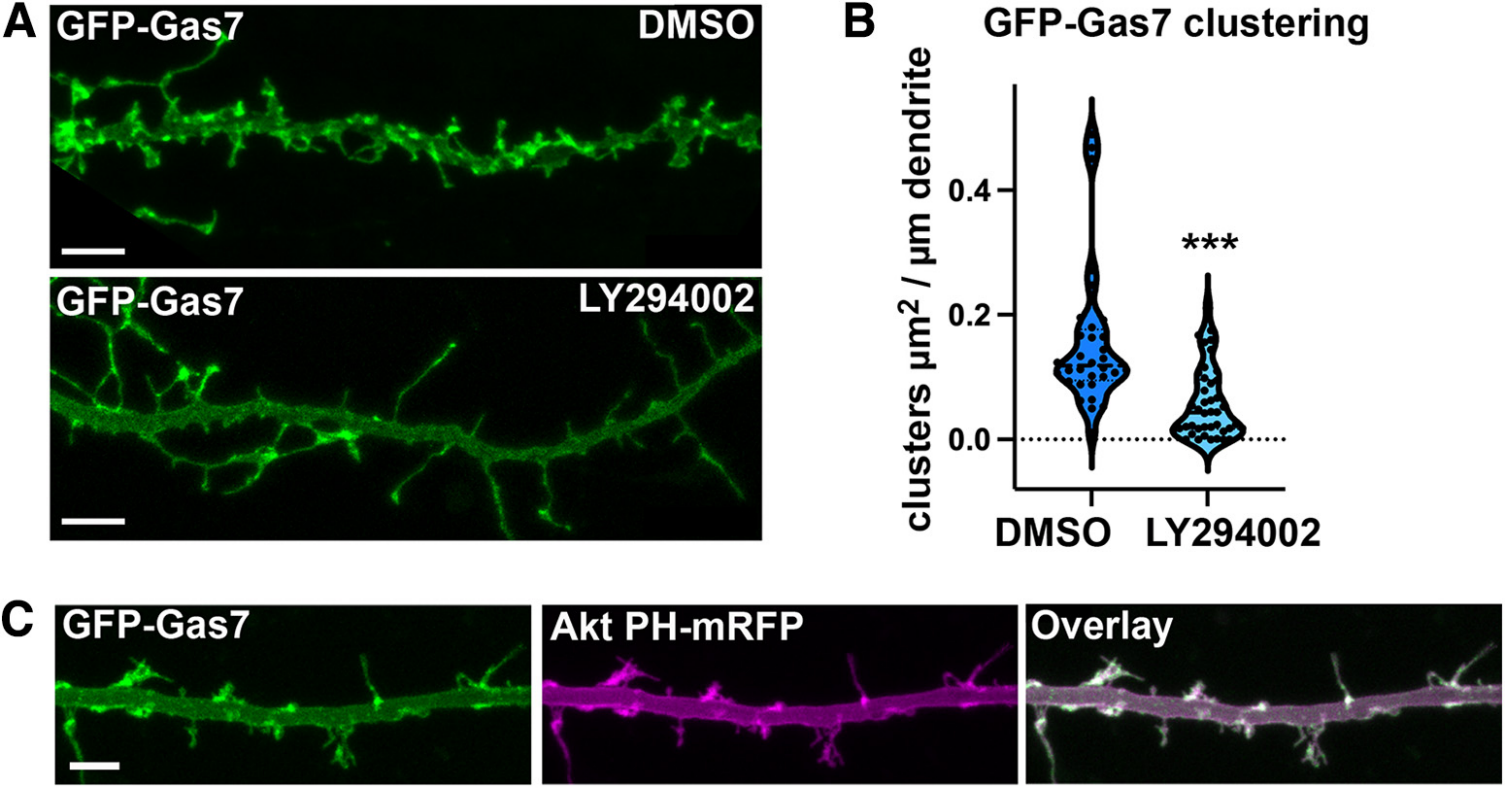
Gas7 clustering requires PI3-kinase activity. ***A***, Dendritic segments of GFP-Gas7-expressing neurons treated with DMSO or 100 μm PI3K-inhibitor LY294002 for 10 min. Scale bars, 5 µm. ***B***, Quantification of GFP-Gas7 clusters in GFP-Gas7-expressing neurons treated with either DMSO or 100 μm LY294002. ***p < 0.001 as determined by Mann–Whitney *U* test. DMSO: *n* = 32 neurons; LY294002: *n* = 42 neurons. GFP-Gas7 cluster area (µm^2^) per dendrite length (µm) was 0.157 ± 0.020 for DMSO-treated Gas7-overexpressing neurons and 0.058 ± 0.009 for LY294002-treated Gas7-overexpressing neurons (mean ± SEM). Violin plot presents the median and interquartile range (25th and 75th percentiles). Data are pooled from three experiments. ***C***, Representative image of a dendritic segment of a neuron imaged live transfected with GFP-Gas7 and Akt PH domain-mRFP. Scale bar, 5 µm. See also Movie 4. See also Extended Data Figure 7-1.

Gas7 binds PI(3,4,5)P3 *in vitro* (Hanawa-Suetsugu et al., 2019). To examine whether the localization of Gas7 to the dendritic membrane and clusters requires PI3-kinase (PI3K) activity, we treated cells expressing GFP-Gas7 with a PI3K inhibitor LY294002 for 10 min. PI3K inhibition significantly reduced the GFP-Gas7 clustering on the dendrites (Fig. 7*A*,*B*). We further followed the effect of LY294002 with time-lapse imaging. From time-lapse videos we counted all new spines before and after LY294002 treatment. This analysis showed that PI3K inhibition reduces the rate of spine formation (Extended Data Fig. 7-1*A*). As expected, the percentage of spines initiated from GFP-Gas7 clusters was also reduced (Extended Data Fig. 7-1*B*). Moreover, we tested whether GFP-Gas7 co-localizes with a PI(3,4,5)P3 marker. PH domain from Akt, which has high specificity for PI(3,4,5)P3 and PI(3,4)P2 (Ebner et al., 2017) exhibited similar localization pattern with GFP-Gas7 in living neurons (Fig. 7*C*; Movie 4).

10.1523/ENEURO.0344-22.2023.f7-1 Extended Data Figure 7-1 Extended data supporting Figure 7. PI3-kinase inhibition reduced the spine initiation rate and percent of spines initiated from GFP-Gas7 clusters. ***A***, Quantification of the rate of formation of new protrusions 10–15 min before and 10–15 min after 100 µm PI3K inhibitor (LY294002) treatment in GFP-Gas7-expressing neurons. The average number of new protrusions/10 µm/1 min before LY294002 treatment was 0.212 ± 0.029 compared to that after LY294002 treatment, 0.088 ± 0.017. *p < 0.05 as determined by Wilcoxon matched-pairs signed-rank test *n* = 7 videos). ***B***, Quantification of the percentage of new protrusions initiating from GFP-Gas7 clusters before and after 100 µm PI3K inhibitor (LY294002) treatment in GFP-Gas7-expressing neurons. The average percentage of new protrusions initiating from GFP-Gas7 clusters before LY294002 treatment was 73.106 ± 4.572 compared to that after LY294002 treatment, 37.800 ± 4.374. *p < 0.05 as determined by Wilcoxon matched-pairs signed-rank test (*n* = 7 videos). (Download Figure 7-1, TIF file.

### Positively charged amino acids in F-BAR domain are necessary for Gas7 localization and functionality

**Figure 8.**
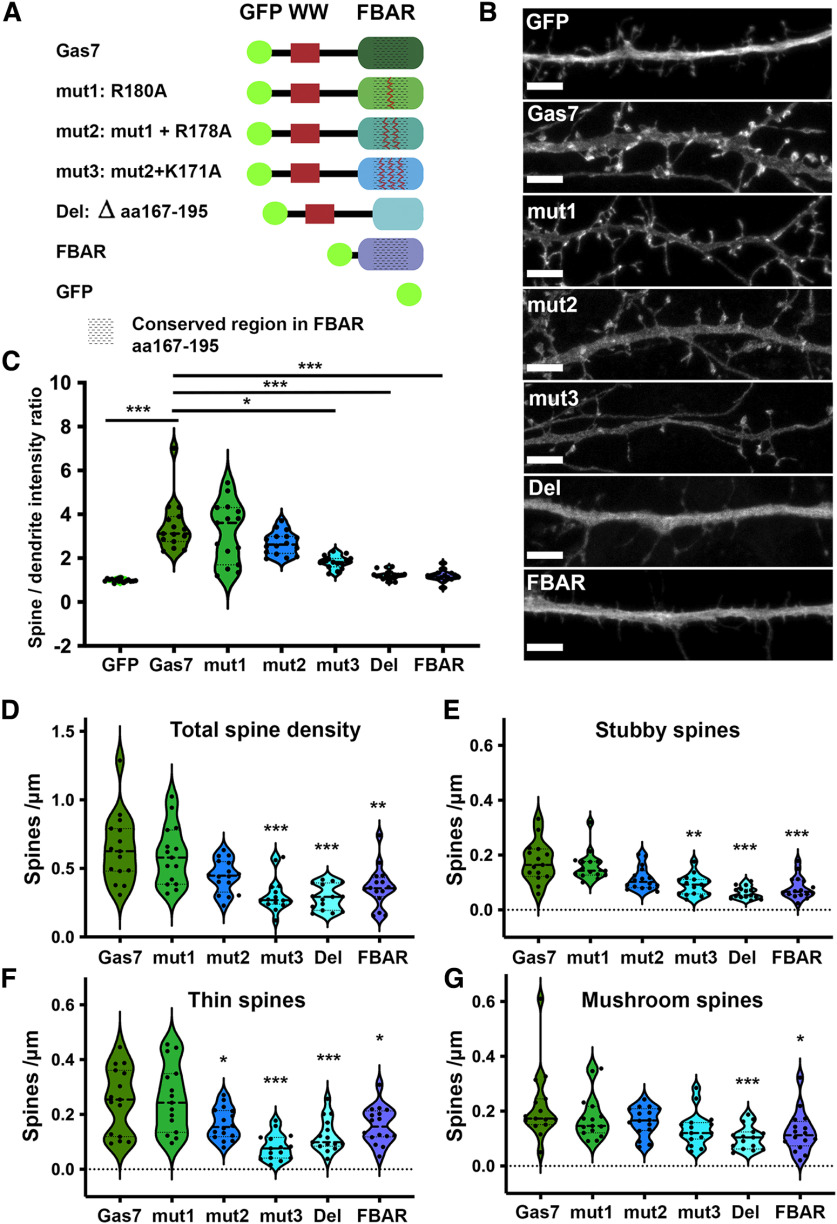
Positively charged amino acids in F-BAR domain are necessary for proper localization and functionality of Gas7. ***A***, Schematic representation of the wild-type and mutant GFP-Gas7 constructs. ***B***, Representative images showing the effect of overexpression of wild-type and mutant GFP-Gas7 constructs in 15 DIV primary hippocampal neurons. Scale bars, 5 µm. See also Extended Data Figure 8-1. ***C***, Quantification of dendritic spine/dendrite intensity ratio analysis for hippocampal neurons overexpressing wild-type and mutant GFP-tagged constructs. The spine/dendrite intensity ratio was 0.99 ± 0.019 for GFP, 3.466 ± 0.302 for Gas7, 3.176 ± 0.358 for mut1, 2.682 ± 0.130 for mut2, 1.82 ± 0.075 for mut3, 1.228 ± 0.047 for Del, and 1.187 ± 0.061 for FBAR. *p < 0.05 and ***p < 0.001 as determined Kruskal–Wallis test (GFP: *n* = 16 neurons; Gas7: *n* = 15 neurons; mut1 = 15 neurons; mut2 = 16 neurons; mut3 = 15 neurons; Del = 15 neurons; FBAR = 16 neurons). Violin plot presents the median and interquartile range (25th and 75th percentiles). ***D–G***, Spine density analysis for all (***D***), stubby spines (***E***), thin spines (***F***), and mushroom spines (***G***). Same dataset used as in ***C***. Comparisons GFP-Gas7 versus mutated GFP-Gas7. Violin plot presents the median and interquartile range (25th and 75th percentiles). ***D***, Quantification of the total spine density. The total spine density was 0.645 ± 0.065 for WT Gas7, 0.595 ± 0.056 for mut1, 0.444 ± 0.030 for mut2, 0.307 ± 0.032 for mut3, 0.290 ± 0.024 for Del, and 0.364 ± 0.037 for FBAR. **p < 0.01 and ***p < 0.001 as determined by Kruskal–Wallis test. ***E***, Quantification of the stubby spine density. The stubby spine density was 0.176 ± 0.019 for WT Gas7, 0.160 ± 0.014 for mut1, 0.115 ± 0.011 for mut2, 0.090 ± 0.010 for mut3, 0.063 ± 0.006 for Del, and 0.081 ± 0.010 for FBAR. **p < 0.01 and ***p < 0.001 as determined by Kruskal–Wallis test. ***F***, Quantification of the thin spine density. The thin spine density was 0.251 ± 0.031 for WT Gas7, 0.258 ± 0.032 for mut1, 0.166 ± 0.014 for mut2, 0.081 ± 0.012 for mut3, 0.124 ± 0.015 for Del, and 0.160 ± 0.017 for FBAR. *p < 0.05 and ***p < 0.001 as determined by one-way ANOVA with Games–Howell *post hoc* test. ***G***, Quantification of the mushroom spine density. The mushroom spine density was 0.217 ± 0.033 for WT Gas7, 0.177 ± 0.021 for mut1, 0.164 ± 0.013 for mut2, 0.136 ± 0.016 for mut3, 0.103 ± 0.011 for Del, and 0.123 ± 0.018 for FBAR. *p < 0.05 and ***p < 0.001 as determined by Kruskal–Wallis test.

We expected that Gas7 binds to the plasma membrane via its F-BAR domain and therefore we mutated positively charged amino acids in the conserved region or deleted the whole conserved region (Fig. 8*A*). When we were building mutant constructs, the Gas7 F-BAR structure was not yet available. Thus, we mutated amino acids located to the first α-helix, which were conserved among different BAR domains and were associated with a loss of lipid-binding activity in other F-BAR domains (Roujeinikova, 2014; Hanawa-Suetsugu et al., 2019). F-BAR domains bind to the negatively charged surface of the lipid bilayer via an extensive positively charged patch on their surface. The mutated original amino acids are all positively charged (K or R) and we neutralized the charge by mutating them to alanine (K171A, R178A, R180A; Fig. 8*A*). We also cloned a construct containing only the F-BAR domain. We then expressed these GFP-tagged mutated proteins in cells (Fig. 8*B*) and compared their subcellular localization to co-expressed mCherry (Extended Data Fig. 8-1). GFP was equally distributed between the dendritic shaft and spines, whereas wild-type Gas7 was 3.5-times more concentrated on spines (Fig. 8*C*). Each single change of a positive charge to neutral charge reduced the localization of mutant proteins to spines (Fig. 8*C*). The mutant with the deletion of the conserved region was comparable to GFP control. F-BAR domain alone looked as diffuse as GFP suggesting that F-BAR domain alone is not sufficient to localize protein. These results show that positively charged amino acids located in the conserved region of Gas7 F-BAR domain are necessary for the proper location of Gas7. However, Gas7 F-BAR is not sufficient for the proper location suggesting that other sequences or domains are required for the proper localization. We then wanted to test whether these F-BAR mutations affect the efficiency of GFP-Gas7 to increase spine density as shown in Figure 2. The single mutation R180A (mut1) did not change wild-type GFP-Gas7 effect on spine density, and double mutation R180A+R178A (mut2) showed difference only in thin spine density, but all other mutations showed differences in almost all spine types compared with wild-type GFP-Gas7 expression (Fig. 8*D–G*). Taken together, F-BAR mutations reduce GFP-Gas7 localization to spines and they decrease the effect of GFP-Gas7 on spine density indicating that either mutations affect both localization and function or then that reduced localization also reduces functionality. Or reduced functionality reduces localization, although this possibility is less probable. F-BAR domain alone cannot localize or increase spine density.

10.1523/ENEURO.0344-22.2023.f8-1 Extended Data Figure 8-1 Extended data supporting Figure 8*B*. Representative images showing the localization and effect of overexpression of wild-type and mutant GFP-Gas7 constructs in 15 DIV primary hippocampal neurons. On left, there is shown mCherry fluorescence. mCherry was used as a reference color for localization and dendritic spine analysis shown in Figure 8. In the middle is shown GFP fluorescence. Each row shows different GFP-Gas7 wild-type or mutant construct, same constructs and pictures shown in Figure 8. Construct details are explained in Figure 8*A* and in Materials and Methods. On right, there is merged image of mCherry (magenta) and GFP (green) channels. mCherry-GFP control shows 100% overlap of two colors, whereas in mCherry-GFP-Gas7 wild type, they clearly separate. mCherry labels strongly the dendrite (diffuse fill), whereas GFP-Gas7 concentrates on clusters and dendritic spines. The more the F-BAR domain has mutations, the less GFP-Gas7 concentrates to clusters and spines. Scale bars, 5 µm. Download Figure 8-1, TIF file.

## Discussion

Neuronal activity regulates the formation of new connections (Papa and Segal, 1996; Engert and Bonhoeffer, 1999; Maletic-Savatic et al., 1999; Kwon and Sabatini, 2011). An obvious mechanism to create new connections is to initiate new spines to reach out to another neuron. Hamilton et al., shed light on the mechanism of activity-induced spine initiation in 2012 by showing the need of proteosomal activity in spine initiation after neuron activation (Hamilton et al., 2012). Seven years later, Chatzi et al. (2019) showed that neuronal activity increases the expression of ABBA/Mtss1l, and this was linked to formation of new spines. Following ideas from this study, we hypothesized that spine-initiating BAR domain proteins could be regulated by neuronal activity.

Spine initiation factors are molecules which facilitate spine initiation. Known spine initiation factors MIM/Mtss1 and SrGAP3 curve the plasma membrane to induce proto-protrusions which help to initiate filopodium growth. Gas7 does not curve the membrane *in vitro*, but it creates flat scaffolds under the plasma membrane (Hanawa-Suetsugu et al., 2019). However, it may prefer curved membrane for membrane binding (Hanawa-Suetsugu et al., 2019). In our experiments, Gas7-induced new protrusions often initiated with a curved plasma membrane (Fig. 2; Extended Data Fig. 2-1). To facilitate spine initiation Gas7 is at right place at right time, just before a new filopodium protrudes out from the membrane (Figs. 1, 2). Its overexpression increases spine density (Fig. 2) and its decreased expression reduces spine density (Fig. 4). Bicuculline treatment increased GFP-Gas7 clustering and spine initiation (Fig. 1). When GFP-Gas7 was partially “washed out” from plasma membrane with PI3K inhibitor, spine initiation was also reduced (Extended Data Fig. 7-1). All these results suggest that Gas7 is a novel spine initiation factor.

Gas7 has several functions in neurons and other cell types. In neurons, Gas7 has been earlier shown to be involved in neurite initiation (You and Lin-Chao, 2010). In non-neuronal cells, Gas7 plays an important role in phagocytosis. It is plausible that Gas7 activates the N-WASP and Arp2/3 complex-involved signaling pathways in these different cellular processes.

Gas7 binds PI(3,4,5)P3 (Hanawa-Suetsugu et al., 2019) and we demonstrated that PI3K activity is needed for the proper localization of GFP-Gas7 (Fig. 7*A*,*B*). Although PI3K inhibition may affect Gas7 localization in neurons also through possible change in spine density and morphology, we believe it is unlikely that changes in morphology per se impact Gas7 clustering as similar change in cell morphology on latrunculin B treatment did not decrease GFP-Gas7 clustering (Fig. 5). We also showed that GFP-Gas7 colocalized with PI(3,4,5)P3/PI(3,4)P2 marker in living neurons (Fig. 7*C*) which indicates that PIPs probably play a role in Gas7 localization and clustering. We further mutated positively charged amino acids from the first α helix of F-BAR domain and showed that mutations reduced the localization of GFP-Gas7 to spines (Fig. 8*C*). Mutations which had biggest reduction in GFP-Gas7 localization to spines, also reduced the GFP-Gas7 overexpression effect on spine density (Fig. 8*D–G*). Thus, it seems that Gas7 binding to PI(3,4,5)P3 is required for the proper localization of Gas7 as well as for its function in increasing spine density. For these mutation experiments, we selected mutated amino acids following the idea of the conserved region, but retrospectively, based on results of Hanawa-Suetsugu et al. (2019), it is expected that there are important amino acids also in other α helixes of F-BAR domain and mutating any of those will possibly reduce binding.

We also analyzed the localization of F-BAR domain alone. MIM/Mtss1 I-BAR domain localized strongly to the plasma membrane and it induced finger-like filopodia all over the dendrites (Saarikangas et al., 2015). SrGAP3 iF-BAR domain could rescue spine phonotype of SrGAP3 deletion (Carlson et al., 2011). In contrast, instead of localizing to plasma membrane or inducing protrusions, Gas7 F-BAR domain exhibited diffuse localization and it failed to mimic spine phenotype induced by wild-type GFP-Gas7 (Fig. 8). Similarly, in a phagocytosis study, the Gas7 F-BAR domain failed to rescue phagocytosis (Hanawa-Suetsugu et al., 2019), suggesting the requirement of the WW domain or other Gas7 sequences. Similar to our experimental results, the membrane binding of Gas7 F-BAR was anyway essential, as the full length Gas7 constructs with membrane binding reducing mutations in the F-BAR domain also failed to restore phagocytosis (Hanawa-Suetsugu et al., 2019). Thus, it seems that membrane binding by F-BAR domain is necessary but not sufficient for Gas7 localization and phenotype.

Earlier results have indicated that the activity-induced spine outgrowth is dependent on NMDA receptor signaling (Engert and Bonhoeffer, 1999; Maletic-Savatic et al., 1999; Kwon and Sabatini, 2011). NMDA receptor signals through PI3K and therefore it is probable that neuronal activation starts a signaling cascade which activates PI3K (Wang et al., 2012; Arendt et al., 2014). PI3K phosphorylates PI(4,5)P2 to PI(3,4,5)P3 which could recruit Gas7 to a certain area on the plasma membrane. Gas7 recruits N-WASP and Arp2/3 complex and together these proteins seem to create a bigger scaffold with polymerizing actin filaments. It seems that these scaffolds increase the probability of initiating a new spine.

Expression of ABBA/Mtss1l has been shown to increase in a mouse brain in response to physical exercise (Chatzi et al., 2019). shRNA-mediated ABBA/Mtss1l knock-down *in vivo* prevented the exercise-induced increase in spine density and EPSCs. In our current study, we showed that Gas7 localization to spine initiation sites can be enhanced by neuron activation (Fig. 1). This evidence suggests that different spine initiation factors are differently regulated by neuronal activity.

Gas7, SrGAP3 and ABBA/Mtss1l have relatively broad expression throughout the mouse life (Khanal and Hotulainen, 2021). In contrast, the expression of MIM/Mtss1 drops after early development in all other brain regions except the Purkinje cells in cerebellum (Minkeviciene et al., 2019). During early development, spine density increases quickly (Huttenlocher, 1979). It might be that during development the spines are added randomly to generate a preliminary neuronal network which can then be defined later in life (Runge et al., 2020). MIM/Mtss1 could serve as an early spine initiation factor, which produces spines efficiently all over the dendrites. After early development, spine initiation could be shifted to activity-based spine initiation and this could be driven by Gas7, ABBA/Mtss1l and possibly other unidentified factors. The exact location of a new spine might not be crucial. Instead, it might be sufficient to increase the probability to initiate new spines to wire the firing neurons together.

## Synthesis

Reviewing Editor: Kristen Harris, The University of Texas at Austin Decisions are customarily a result of the Reviewing Editor and the peer reviewers coming together and discussing their recommendations until a consensus is reached. When revisions are invited, a fact-based synthesis statement explaining their decision and outlining what is needed to prepare a revision will be listed below. The following reviewer(s) agreed to reveal their identity: Karen Zito, Sergei Kirov. Note: If this manuscript was transferred from JNeurosci and a decision was made to accept the manuscript without peer review, a brief statement to this effect will instead be what is listed below.

The authors have now provided a revised manuscript describing the role of Gas7 in activity-dependent dendritic spine formation. The authors have done an excellent job thoroughly addressing many points; however, a few items remain insufficiently addressed. We retain the numbering from the rebuttal and discuss a few of these items that need further attention.

TITLE: Considering the title of the paper and the argument that Gas7 plays a role in activity-dependent spine outgrowth, it is not clear why the authors do not show that bicuculline-induced new outgrowth (Fig 1E) is inhibited by RNAi of Gas7. Otherwise, the relationship is only correlative. A title adjustment referring to regulation of spine density rather than activity-dependent spine outgrowth could be an alternative way of addressing this issue without further experiments and then of course current model can still be proposed as a model of Gas7 action.

Editor: - Either do the suggested controls - or change the title. This issue can not be ignored.

Specific Points that must be addressed:

1. The authors updated several figure panels to designate GFP-Gas7 when utilized, as requested; however, many were not updated. Please update Figs. 1D, 1F, 2E, 2F, 5C, 5D merge, 5E, 6A merge, 6C, 6D, 6F, 6G, 7B, 8A, 8B, 8C, 8D, 8E, 8F, 8G. Also, Figs. 2G, 2H should indicate mCherry-Gas7. Importantly, the text and Figure legends also need to be updated from “Gas7” to state “GFP-Gas7” when referring to GFP-Gas7. Finally, all Supp Figs and figure legends should also be adjusted to indicate GFP-Gas7 and mCherry-Gas7, when appropriate.

2. A vehicle control is standard in any experiment with a drug application, as in Fig 1, because the abrupt addition of even water alone could cause changes to occur (via osmotic changes) independent of drug. In this case, previous publications have shown that bicuculline enhances spine outgrowth, so that aspect at least should be acceptable. An additional concern is that a sample size of 4 cells seems very low (evident in the size of the error bars!). Finally, in addition to (or instead of) number of independent brain slices, the number of independent brain slice preparations (animals) should be indicated.

12. In Fig 8 (previously part of Fig 7), the authors have chosen to continue to present the two channels separately rather than together, which is understandable. However, in that case, please provide the red channel images that were used for spine counting in a supplemental figure.
