# Supplementary figures and images for: Gas7 Is a Novel Dendritic Spine Initiation Factor
Source: eNeuro. 2023 Apr 13;10(4):ENEURO.0344-22.2023. doi: 10.1523/ENEURO.0344-22.2023 (PMC10114493; doi:10.1523/ENEURO.0344-22.2023)

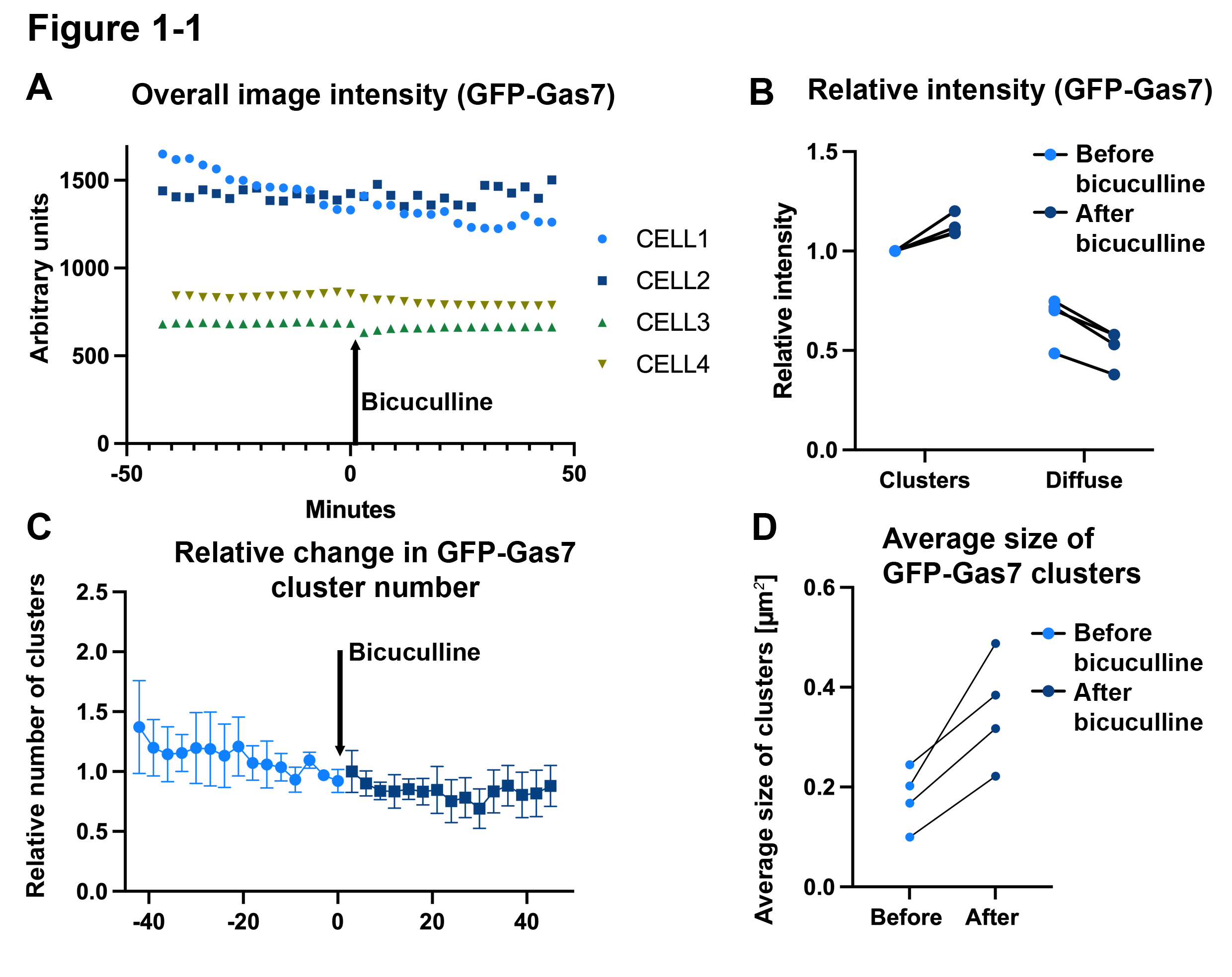

Supplement: Extended Data Figure 1-1 — Extended reporting of cluster analysis of bicuculline-treated GFP-Gas7-expressing pyramidal neurons supporting Figure 1C,D. A, Overall image intensity of the analyzed videos. Frames are acquired every 3 min and there are 15 frames before and after 30 µm bicuculline treatment. Overall intensity decreases in one cell, otherwise intensities are relatively stable. All analyses of the figure are from same dataset containing four GFP-Gas7-expressing pyramidal neurons each from independent organotypic slice. B, Relative GFP-Gas7 fluorescence intensity in clusters and noncluster areas. GFP-Gas7 fluorescence intensity increases in all analyzed cells in cluster areas and it decreases in noncluster areas after adding bicuculline. Intensities are relative intensities so that intensity in clusters before bicuculline is set to 1. C, Relative number of clusters slowly decreases during imaging. Addition of bicuculline did not show any change to gradual decrease. The relative number of clusters is calculated by dividing each of the original number of clusters by the average number of clusters obtained from the last three frames before adding bicuculline. D, Average size of clusters before and after bicuculline treatment in µm2. Before is average of the last three frames before bicuculline treatment. After is highest average value in first 10 min after bicuculline treatment. Thus, this is the maximum change in cluster size. On average, cluster size doubles. Download Figure 1-1, TIF file. [file enu-eN-NWR-0344-22-s02.tif]

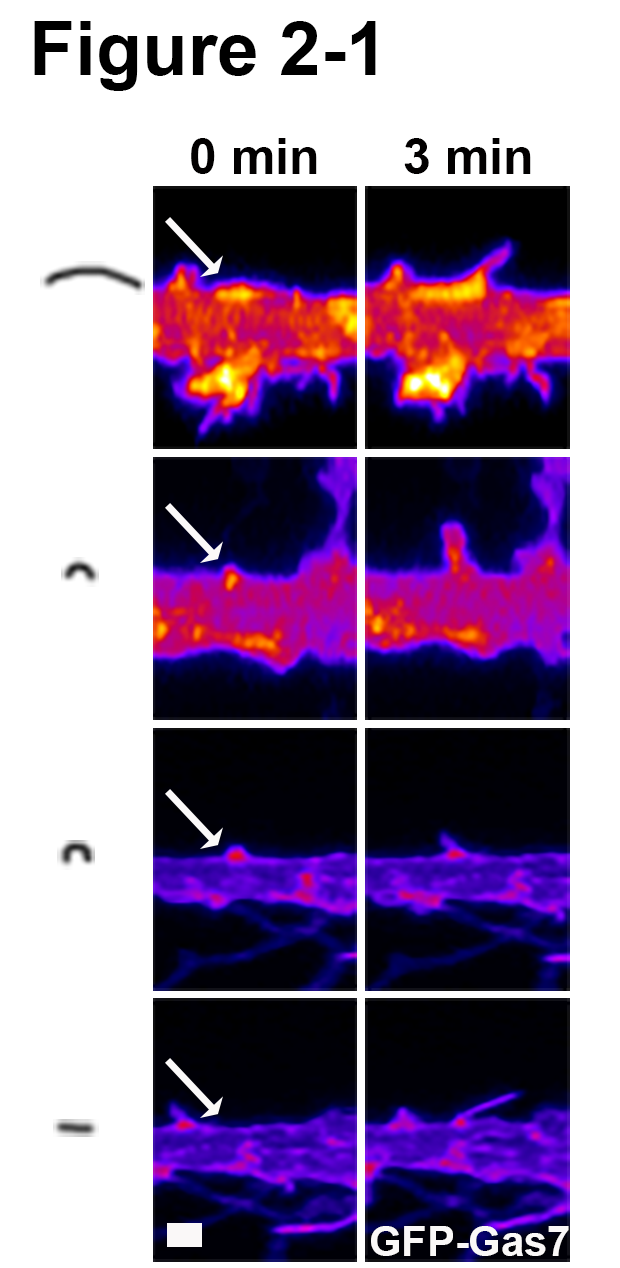

Supplement: Extended Data Figure 2-1 — Extended data supporting Figure 2F. GFP-Gas7-associated membrane curvatures preceding new filopodia formation. Time frames of a segment of hippocampal neurons transfected with GFP-Gas7. New filopodia often emerged from GFP-Gas7 clusters with different membrane curvatures. Curvature is visualized with drawn line on left. Time frames are 3 min apart from each other. Arrows point to areas were new filopodia form. Scale bar, 1 μm. Pseudocolored with intensity-based Fire. Download Figure 2-1, TIF file. [file enu-eN-NWR-0344-22-s03.tif]

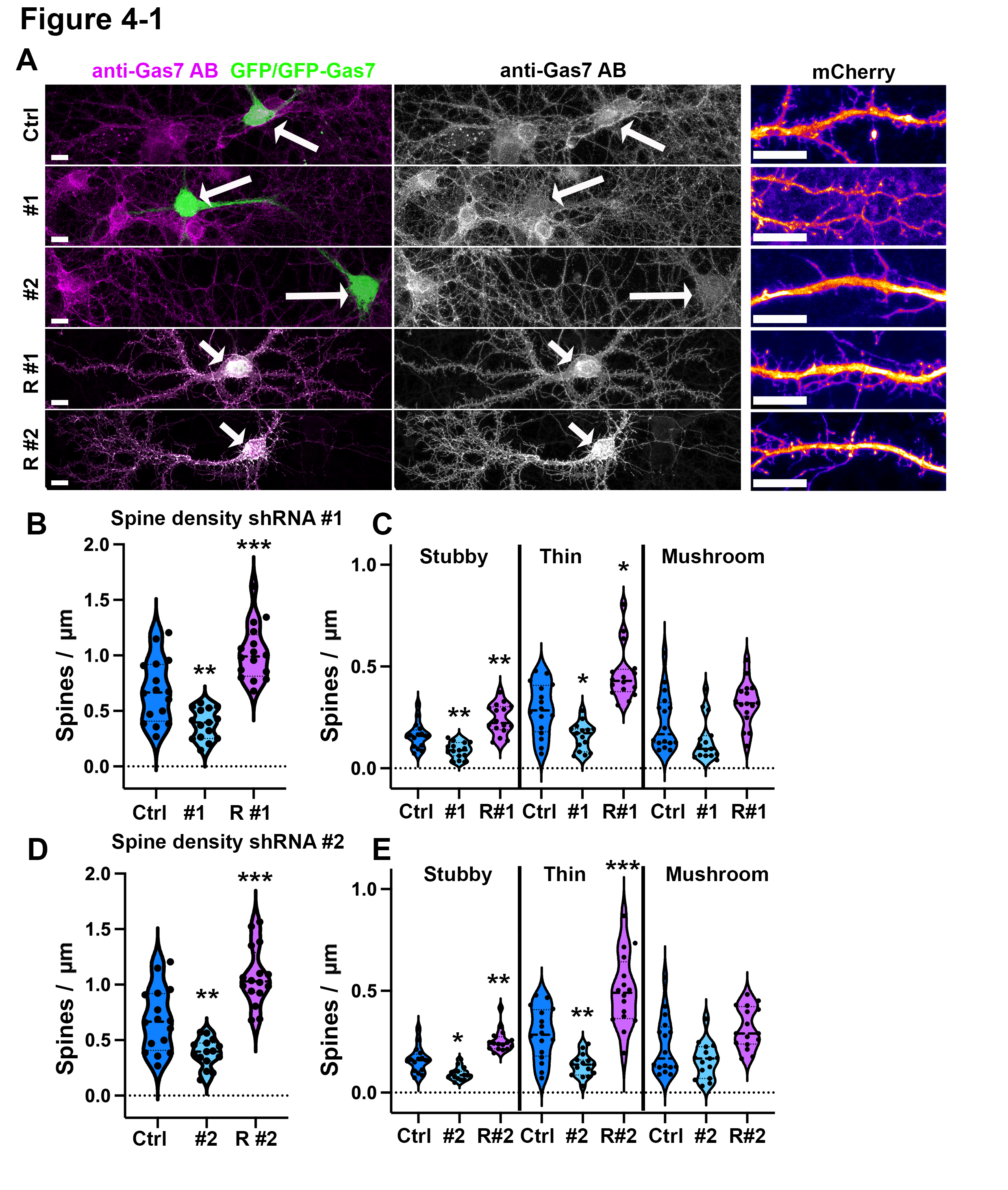

Supplement: Extended Data Figure 4-1 — Extended data supporting Figure 4. Human GFP-Gas7 overexpression rescues Gas7 knock-down induced spine phenotype. A, Representative dendritic segments of hippocampal neurons expressing mCherry together with scrambled shRNA (ctrl), Gas7 shRNA #1 (#1 and R #1), or Gas7 shRNA #2 (#2 and R #2). ctrl, #1 and #2 are also transfected with GFP. In R #1 and R #2, knock-down of endogenous Gas7 is rescued by overexpressing human GFP-Gas7, which is resistant to used shRNAs. All cells are stained with anti-Gas7 antibody. mCherry channel was used for analyzing dendritic spine density and morphology (B). mCherry on right is shown as intensity-based pseudocoloring (Fire). All scale bars, 10 µm. On left, is shown anti-Gas7 antibody staining in magenta and either GFP or GFP-Gas7 in green. In the middle, anti-Gas7 antibody staining is shown in black and white to see different expression levels in transfected cell somas compared to neighboring nontransfected cells. In control cells transfected with scrambled shRNA, Gas7 staining is at similar level compared to nontransfected cells. In Gas7 shRNA-transfected, but not rescued, cells (#1 and #2), Gas7 expression is lower than in neighboring cells. In cells rescued with GFP-Gas7 expression (R #1 and R #2), Gas7 level is higher than in neighboring cells and transfected cells are easy to distinguish from nontransfected cells. White arrows point to somas of transfected cell somas. mCherry images on right (higher zoom in of cells shown on left) show that Gas7 shRNA-transfected cells have less spines compared to controls or rescued cells. B, Quantification of total dendritic spine density for neurons expressing scrambled shRNA (ctrl), Gas7 shRNA #1 (#1), and Rescued shRNA #1 (R #1). The total spine density was ctrl = 0.68 ± 0.071, #1 = 0.38 ± 0.036, R #1 = 1.02 ± 0.062. Ctrl: n = 16 neurons; #1: n = 15 neurons; R #1 16 neurons. Data is pooled from three experiments and represented as mean ± SEM. **p < 0.01 and ***p < 0.001 as determined by one- [file enu-eN-NWR-0344-22-s04.tif]

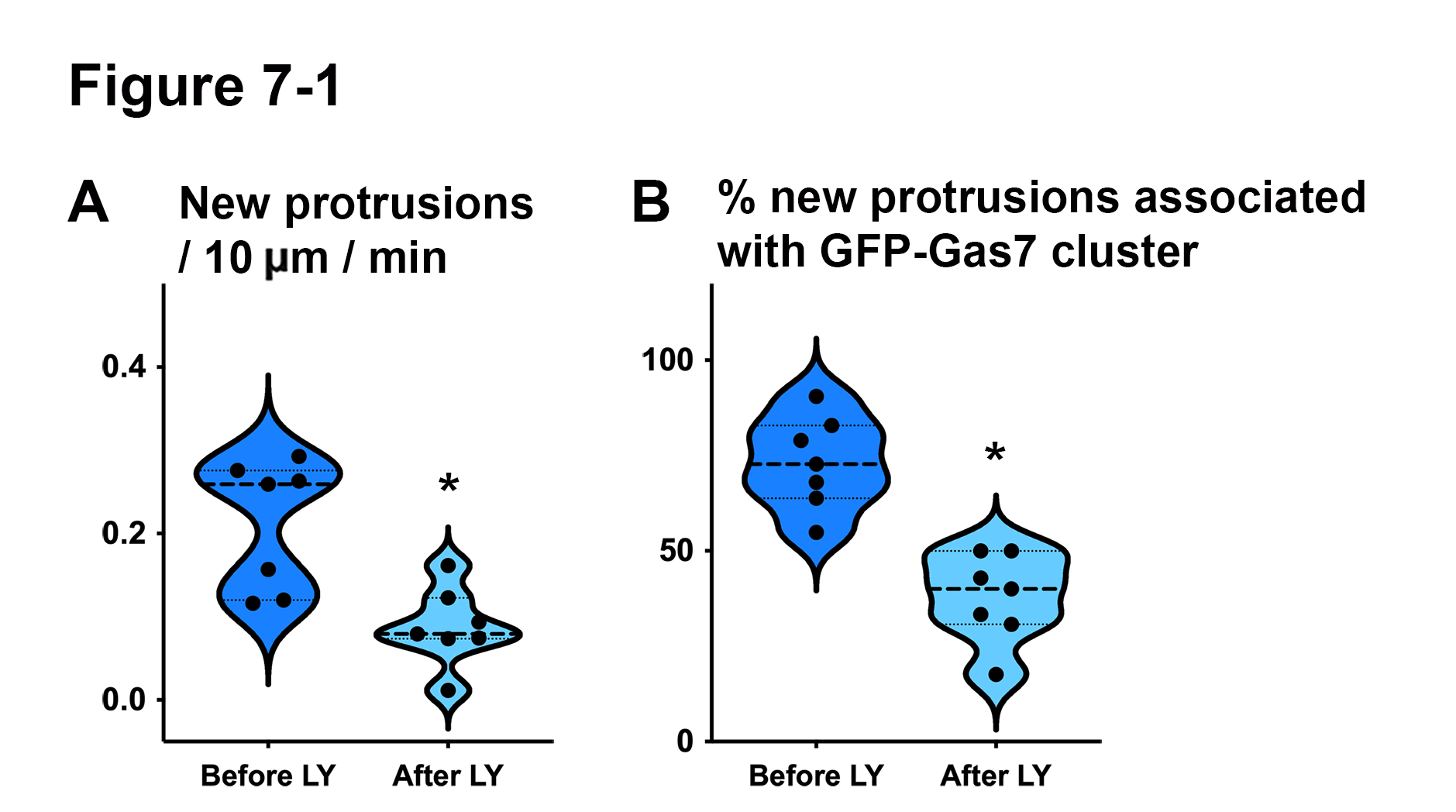

Supplement: Extended Data Figure 7-1 — Extended data supporting Figure 7. PI3-kinase inhibition reduced the spine initiation rate and percent of spines initiated from GFP-Gas7 clusters. A, Quantification of the rate of formation of new protrusions 10–15 min before and 10–15 min after 100 µm PI3K inhibitor (LY294002) treatment in GFP-Gas7-expressing neurons. The average number of new protrusions/10 µm/1 min before LY294002 treatment was 0.212 ± 0.029 compared to that after LY294002 treatment, 0.088 ± 0.017. *p < 0.05 as determined by Wilcoxon matched-pairs signed-rank test n = 7 videos). B, Quantification of the percentage of new protrusions initiating from GFP-Gas7 clusters before and after 100 µm PI3K inhibitor (LY294002) treatment in GFP-Gas7-expressing neurons. The average percentage of new protrusions initiating from GFP-Gas7 clusters before LY294002 treatment was 73.106 ± 4.572 compared to that after LY294002 treatment, 37.800 ± 4.374. *p < 0.05 as determined by Wilcoxon matched-pairs signed-rank test (n = 7 videos). (Download Figure 7-1, TIF file. [file enu-eN-NWR-0344-22-s05.tif]

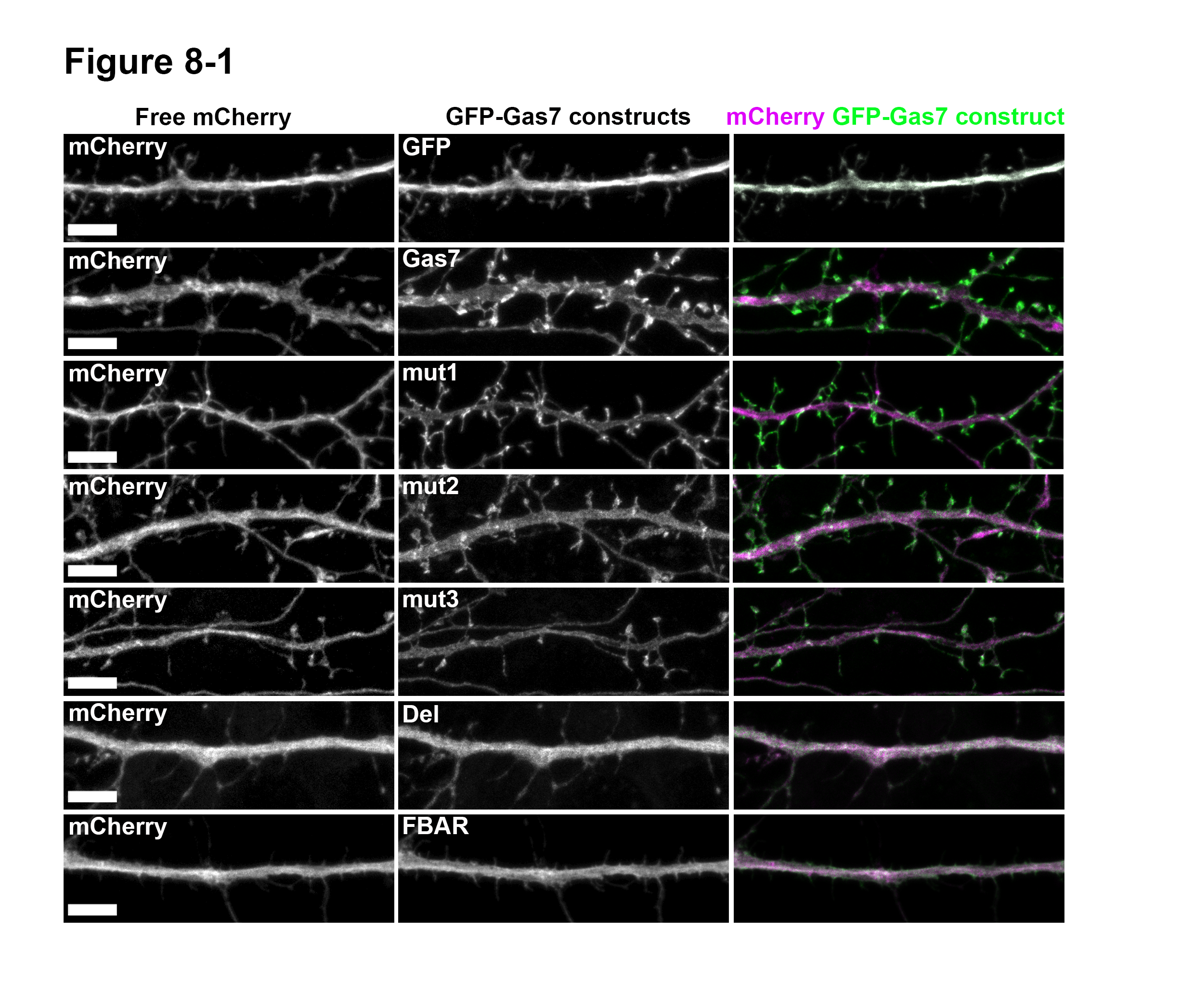

Supplement: Extended Data Figure 8-1 — Extended data supporting Figure 8B. Representative images showing the localization and effect of overexpression of wild-type and mutant GFP-Gas7 constructs in 15 DIV primary hippocampal neurons. On left, there is shown mCherry fluorescence. mCherry was used as a reference color for localization and dendritic spine analysis shown in Figure 8. In the middle is shown GFP fluorescence. Each row shows different GFP-Gas7 wild-type or mutant construct, same constructs and pictures shown in Figure 8. Construct details are explained in Figure 8A and in Materials and Methods. On right, there is merged image of mCherry (magenta) and GFP (green) channels. mCherry-GFP control shows 100% overlap of two colors, whereas in mCherry-GFP-Gas7 wild type, they clearly separate. mCherry labels strongly the dendrite (diffuse fill), whereas GFP-Gas7 concentrates on clusters and dendritic spines. The more the F-BAR domain has mutations, the less GFP-Gas7 concentrates to clusters and spines. Scale bars, 5 µm. Download Figure 8-1, TIF file. [file enu-eN-NWR-0344-22-s10.tif]
